# Chromosome assembly of large and complex genomes using multiple references

**DOI:** 10.1101/gr.236273.118

**Published:** 2018-11

**Authors:** Mikhail Kolmogorov, Joel Armstrong, Brian J. Raney, Ian Streeter, Matthew Dunn, Fengtang Yang, Duncan Odom, Paul Flicek, Thomas M. Keane, David Thybert, Benedict Paten, Son Pham

**Affiliations:** 1 Department of Computer Science and Engineering, University of California, San Diego, California 92093, USA;; 2 Center for Biomolecular Science and Engineering, University of California, Santa Cruz, California 95064, USA;; 3 European Molecular Biology Laboratory, European Bioinformatics Institute, Wellcome Genome Campus, Hinxton CB10 1SD, United Kingdom;; 4 Wellcome Sanger Institute, Wellcome Genome Campus, Hinxton CB10 1SA, United Kingdom;; 5 Cancer Research UK Cambridge Institute, University of Cambridge, CB2 0RE Cambridge, United Kingdom;; 6 School of Life Sciences, University of Nottingham, Nottingham NG7 2NR, United Kingdom;; 7 Earlham Institute, Norwich Research Park, Norwich NR4 7UG, United Kingdom;; 8 BioTuring Incorporated, San Diego, California 92121, USA

## Abstract

Despite the rapid development of sequencing technologies, the assembly of mammalian-scale genomes into complete chromosomes remains one of the most challenging problems in bioinformatics. To help address this difficulty, we developed Ragout 2, a reference-assisted assembly tool that works for large and complex genomes. By taking one or more target assemblies (generated from an NGS assembler) and one or multiple related reference genomes, Ragout 2 infers the evolutionary relationships between the genomes and builds the final assemblies using a genome rearrangement approach. By using Ragout 2, we transformed NGS assemblies of 16 laboratory mouse strains into sets of complete chromosomes, leaving <5% of sequence unlocalized per set. Various benchmarks, including PCR testing and realigning of long Pacific Biosciences (PacBio) reads, suggest only a small number of structural errors in the final assemblies, comparable with direct assembly approaches. We applied Ragout 2 to the *Mus caroli* and *Mus pahari* genomes, which exhibit karyotype-scale variations compared with other genomes from the *Muridae* family. Chromosome painting maps confirmed most large-scale rearrangements that Ragout 2 detected. We applied Ragout 2 to improve draft sequences of three ape genomes that have recently been published. Ragout 2 transformed three sets of contigs (generated using PacBio reads only) into chromosome-scale assemblies with accuracy comparable to chromosome assemblies generated in the original study using BioNano maps, Hi-C, BAC clones, and FISH.

The year 2001 marked an important step in genome biology with the release of the first near-complete human genome (Lander et al. 2001; Venter et al. 2001). Since then, numerous near-complete mammalian genome sequences have been made available (Pontius et al. 2007; Church et al. 2009; Scally et al. 2012). These finished genomes, while being expensive to produce, have greatly advanced the field of comparative genomics and provided many new insights to our understanding of mammalian evolution. The initial achievement was quickly followed by the era of high-throughput sequencing technologies—next-generation sequencing (NGS). These cost-effective technologies are allowing many sequencing consortia to explore genomes from a large number of species (Jarvis et al. 2014).

Recently, new de novo assembly algorithms have been developed to combine high-throughput short-read sequencing data with long single-molecule sequencing reads or jumping libraries to completely assemble bacterial genomes (one chromosome into one contig) (Koren and Phillippy 2015). In contrast, the complete assembly of mammalian genomes using current short-read sequencing technologies remains a formidable problem, since such genomes are larger and have more complicated repeat structures. Most current mammalian assemblies produced by NGS assemblers (Butler et al. 2008; Simpson et al. 2009) contain thousands to hundreds of thousands of contigs/scaffolds and provide limited value for comparative genomics as constructed syntenic regions are highly fragmented. Recently, some studies (Chaisson et al. 2015; Jain et al. 2018; Kronenberg et al. 2018) have applied long-range technologies (10× Genomics, Hi-C, BioNano, Pacific Biosciences [PacBio], Oxford Nanopore) to improve the assembly of larger genomes. However, the cost of generating long-range connection data in a high-throughput manner is still much higher compared to that of generating short-read libraries.

Since many complete genomes are now available, an alternative approach is to use these genomes to guide the assembly of the target (assembled) genome, in a method called “reference-assisted assembly” (Gnerre et al. 2009). In such methods, the information from a closely related reference genome is used by an NGS assembler for resolving complicated genomic structures, such as repeats or low-coverage regions. This technique was implemented in a number of assemblers/scaffolders (Zerbino and Birney 2008; Peng et al. 2010; Gnerre et al. 2011; Iqbal et al. 2012) and proved to be valuable when a close reference is available. Another common approach is to align preassembled contigs of the target genome against the reference and order them according to their positions in the reference genome (Richter et al. 2007; Rissman et al. 2009). However, for both approaches, simplistic modeling, in which each breakpoint is treated independently, still introduces many misassembly errors when structural variations between the reference and target genomes are present.

To improve over the single-reference-genome approach, Kim et al. (2013) introduced the RACA tool, which represented an important step toward reliable reconstruction of the target genome by analyzing the structure of multiple outgroup genomes in addition to a single reference. The investigators showed that consistent adjacency information across multiple outgroups is a more powerful predictor of adjacencies in the target assembly than single genomes alone. Similar to other genome rearrangement approaches, RACA relies on the decomposition of the input sequences into a set of synteny blocks—long and conservative genomic regions with respect to micro-rearrangements. However, RACA reconstructs synteny blocks by aligning all input sequences against a single reference genome. This approach is biased toward the reference genome and, in some cases, cannot detect synteny blocks (Pham and Pevzner 2010).

To address some limitations of the RACA approach, we previously developed the Ragout algorithm for reference-assisted assembly of bacterial genomes (Kolmogorov et al. 2014), which was coupled with Sibelia (Minkin et al. 2013) for synteny block reconstruction. While we showed that Ragout performed better than RACA on bacterial data sets, Ragout could not be applied to more complex eukaryotic genomes, as the current version of Sibelia is limited to small, closely related genomes with low repeat content. The current mammalian genome assembly strategies typically include multiple stages of scaffolding using different technologies (such as mate-pairs or Hi-C). Aggressive scaffolding strategies typically lead to significant number of errors (Salzberg et al. 2012; Bradnam et al. 2013), which marks another challenge in reference assembly.

This work attempts to address the issues described above. First, we present a new algorithm for synteny block reconstruction for multiple mammalian genomes. Our approach combines Cactus, a multiple whole-genome aligner (Paten et al. 2011), with a new iterative graph simplification algorithm that produces hierarchical synteny blocks on multiple scales. Second, we show how to apply the two-break rearrangement model (Alekseyev and Pevzner 2009) to distinguish between target-specific rearrangements and chimeric misassemblies. We also describe an additional algorithm that fills assembly gaps with missing repetitive sequence by analyzing repeat content of reference genomes. These new algorithms were combined into the Ragout 2 package.

## Results

### Synteny blocks

**Figure 1.**
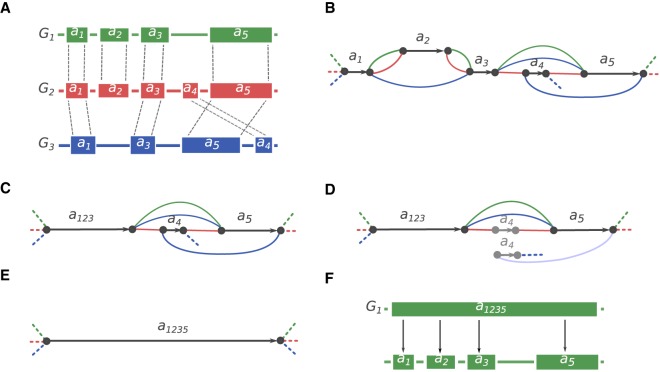
Synteny blocks reconstruction algorithm. (*A*) Three genomes—*G*_*1*_, *G*_*2*_, *G*_*3*_—are encoded in the alphabet of local sequence alignment blocks. |*a*_*4*_| < |*a*_*1*_| < |*a*_*3*_| < |*a*_*2*_| < |*a*_*5*_|. Alignment is performed using the Cactus multiple whole-genome aligner. (*B*) The A-Bruijn graph constructed with *minBlock* = |*a*_*4*_|. Black edges correspond to the alignment blocks, and the colored edges connect the adjacent alignment blocks in the corresponding genome. Dashed edges denote the start/end of each genome. Because the *minBlock* parameter is equal to the size of the smallest block, all blocks were included in the graph. (*C*) The A-Bruijn graph after bubble simplification and collapsing unbranching paths. *a*_*123*_ now represents the new merged block (from *a*_*1*_, *a*_*2*_, *a*_*3*_). (*D*) A next iteration of the A-Bruijn graph with |*a*_*4*_| < *minBlock* ≤ |*a*_*1*_|, which eliminates block *a*_*4*_. (*E*) *a*_*123*_ and *a*_*5*_ are merged into a larger block *a*_*1235*_. (*F*) The hierarchical representation of synteny blocks: The larger block *a*_*1235*_ from the genome *G*_*1*_ can be decomposed into smaller blocks *a*_*1*_, *a*_*2*_, *a*_*3*_, and *a*_*5*_.

Nucleotide-level alignments between diverged genomes contain millions of small variations. To analyze karyotype-level rearrangements, studies typically use lower-resolution mappings. Such mappings are denoted as a set of coarse synteny blocks and could be defined as nonoverlapping strand-oriented chromosome intervals in the set of genomes being compared (Kent et al. 2003; Pevzner and Tesler 2003b). Synteny blocks are a convenient way to represent the homology relationship between large segments of the genomes. In this study, we also use synteny blocks to separate large structural variations from small polymorphisms. However, we take a hierarchical approach, with multiple sets of synteny blocks, each defined at a different resolution, from the coarsest, karyotype level all the way down to the fine-grained, nucleotide level. To create the hierarchy, we use the principles developed by the Sibelia tool (Minkin et al. 2013) but adapted with a graph simplification algorithm for constructing synteny blocks from a multiple genome alignment in HAL format (Hickey et al. 2013), produced by Cactus (Paten et al. 2011). In contrast to other whole-genome aligners, Cactus represents the alignments as nonoverlapping blocks. Our algorithm starts from these local alignment blocks (or synteny blocks of the highest resolution) and constructs an A-Bruijn graph from them (Fig. 1A–F). The graph is then iteratively simplified by removing bubbles and collapsing unbranching paths. As a result, initial coarse blocks are merged into the larger blocks, which defines the hierarchy. See the Methods section “Construction of synteny blocks” for the detailed description of the algorithm.

### Incomplete multicolor breakpoint graph

**Figure 2.**
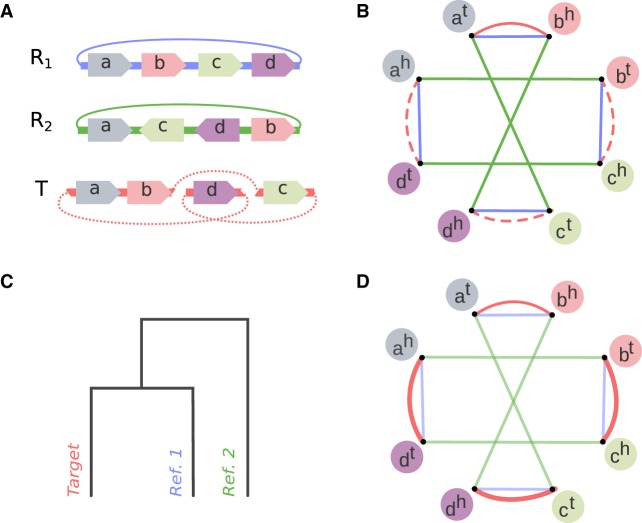
Incomplete breakpoint graph and missing adjacencies inference. (*A*) Two reference genomes (*R*_*1*_ and *R*_*2*_) and a target genome *T* represented in the alphabet of four signed synteny blocks (*a*, *b*, *c*, *d*). All genomes are circular. Reference *R*_*1*_ could be transformed into *R*_*2*_ with two inversions: (*a*, *b*, *c*, *d*) → (*a*, −*c*, −*b*, *d*) → (*a*, −*c*, −*d*, +*b*). Target genome *T* is structurally similar to *R*_*1*_; however, *T* is fragmented into three contigs (missing adjacencies are shown with dashed lines). (*B*) Incomplete breakpoint graph of all genomes. Each synteny block corresponds to two nodes representing its head and tail (denoted as *h* and *t*). Colored edges connect synteny block ends that are adjacent within a corresponding genome. Two inversions between *R*_*1*_ and *R*_*2*_ correspond to two cycles of length four with alternating colors. Because *T* is fragmented, some red adjacencies are missing (dashed lines). (*C*) A phylogenetic tree is reconstructed based on pairwise breakpoint distance between the genomes. Because *T* shares more breakpoints with *R*_*1*_ than with *R*_*2*_, *T* is closer to *R*_*1*_ within the tree. (*D*) Missing adjacencies are recovered based on the graph structure and phylogenetic tree. Since *T* is closer to *R*_*1*_, the algorithm prefers the pair of breakpoints (*b*^*t*^, *c*^*h*^), (*d*^*t*^, *a*^*h*^) to an alternative, (*b*^*t*^, *a*^*h*^), (*c*^*h*^, *d*^*t*^). The reconstructed adjacencies define the synteny block order in the final chromosomes.

The input genome sequences are now represented in the alphabet of synteny blocks. Ragout 2 further constructs an incomplete multicolor breakpoint graph as previously described by Kolmogorov et al. (2014). Here we provide a brief summary of the approach. For the sake of simplicity, let us assume that every synteny block is represented exactly once in each genome and all reference genomes are complete (the issue of repetitive synteny blocks will be addressed later in this paper). Given an assembly *T* and *k* reference sequences *R*_*1*_, … , *R*_*k*_ in the alphabet of synteny blocks *B*, we construct the incomplete multicolor breakpoint graph *BG*(*T*, *R*_*1*_, … , *R*_*k*_) = (*V*, *E*), where *V* = {*b*^*h*^_*i*_, *b*^*t*^_*i*_|*b*_*i*_∈B} (Fig. 2A,B). For each synteny block, there are two vertices in the graph which correspond to the tail and head of the block. Edges are undirected and colored by *k* + 1 colors. An edge connects vertices that correspond to heads/tails of adjacent synteny blocks and is colored by the corresponding color of the genome/assembly. We use *red*, *R*_*1*_, … , *R*_*k*_ to refer to the colors of edges, where red edges represent the adjacencies of synteny blocks in the target assembly *T*, and *R*_*i*_ represents the adjacencies of synteny blocks in genome *R*_*i*_. If the target genome were complete, the set of all red edges would define a perfect matching in the graph. However, since the genome is fragmented into contigs, the adjacency information at the vertices that correspond to contigs ends is missing. The ultimate goal of Ragout 2 is to infer these missing red edges in the graph, which will define the final assembled chromosomes.

### Repeat resolution

As noted above, the breakpoint graph analysis requires all synteny blocks to appear exactly once in each genome. A common approach is to filter out all repetitive blocks before analyzing the breakpoint graph. While this approach works for bacterial genomes, it is not optimal for mammalian assemblies since the number of repeats is much larger. Filtering out all such repeats would lead to many gaps in the final assembly. Here we present a new algorithm that addresses this issue by resolving repeat sequences based on the information from the references. Intuitively, for each unresolved repeat in the target genome, we create *k* different copies of it (where *k* is the estimated copy number of this repeat in the complete genome). As some repeats could already be resolved by the NGS assembler, the corresponding synteny blocks in the target contigs will be surrounded by other unique synteny blocks. We use this “context” information to map repeat instances in contigs to the corresponding repeats in reference genomes. See additional details about the repeat resolution algorithm in the Methods section “Repeat resolution algorithm.”

### Detection of chimeric sequences

In contrast to bacterial assemblies, mammalian assemblies usually contain a higher fraction of misassembled contigs/scaffolds due to increased size and repeat complexity (Salzberg et al. 2012; Bradnam et al. 2013). A large portion of misassembled contigs are chimerical, created when an NGS assembler artificially joins regions in the assembled genome that are not truly adjacent. These false adjacencies correspond to erroneous red edges in the breakpoint graph. Ragout 2 identifies erroneous red edges and removes them before the missing adjacency inference step, since misassemblies in different contigs will join together and cause large structural errors. The Methods section “Detection of chimeric adjacencies” introduces a new algorithm for the detection of erroneous red edges.

### Generating chromosome assemblies

After constructing the initial breakpoint graph, Ragout 2 resolves repeats and removes chimeric connections as described above. Ragout 2 further infers the phylogenetic tree of all genomes (Fig. 2C) by computing pairwise breakpoint distances and applying the neighbor-joining algorithm (Saitou and Nei 1987). In contrast, the original Ragout package required the tree as input, which is not always available (see the Methods section “Phylogenetic tree reconstruction” for the algorithm details). Afterward, Ragout 2 infers missing breakpoint graph adjacencies by solving the half-breakpoint parsimony problem as described by Kolmogorov et al. (2014) (Fig. 2D). The chromosome assemblies are then generated according to the inferred adjacencies. The assembly procedure is repeated in multiple iterations on a different synteny blocks scale, and the final scaffolds are generated as a consensus of multiple iterations. Intuitively, the large synteny block scale provides a reliable “skeleton,” while the smaller synteny blocks help to fill the gaps in assembly (for details, see the Methods section “Iterative assembly”).

### Benchmarking Ragout 2 and RACA on simulated data sets

To benchmark Ragout 2 and RACA performance in the presence of extensive structural rearrangements, we generated multiple data sets from human Chromosome 14 sequence as described in the Methods section “Comparing Ragout 2 and RACA using simulated human Chromosome 14 assemblies.” Each data set included four genomes: three references and a target genome fragmented into contigs (approximately 5000 fragments with a mean length of 18,000 bp). Each data set contained multiple genome rearrangements (from 50 to 500) that were evenly distributed along the branches of the phylogenetic tree. The contigs additionally included 5% of chimeric sequence. To benchmark the Ragout 2 chimera detection module, we performed extra Ragout 2 runs with the following modifications. The first extra run is called *permissive* with the chimera detection module turned off. The second extra run is denoted as *conservative*, in which all unsupported target adjacencies are broken (to mimic the common mapping approach in reference-guided assembly). Note that the permissive strategy benchmark could be also viewed as a comparison of Ragout 2 with the original Ragout, as the original version was not capable for chimera detection. We performed RACA runs on simulated data sets without using read mapping information (similar to the strategy described by Kim et al. 2013) to focus on comparison between the rearrangement models.

**Figure 3.**
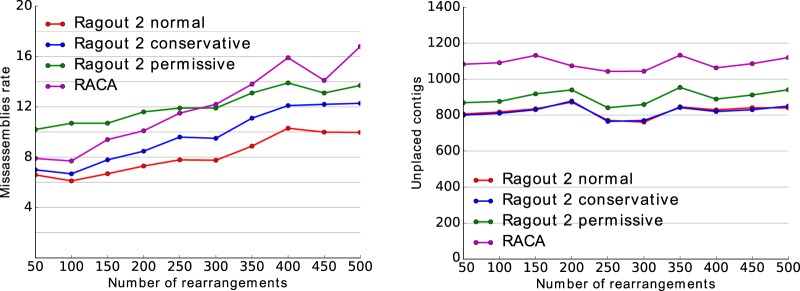
Benchmarking RACA and different Ragout 2 strategies using the simulated human Chr 14 assembly. A target and three reference genomes were simulated by randomly distributing the corresponding number of rearrangements along the branches of a phylogenetic tree. In addition to the normal Ragout 2 run, we performed the “permissive” run in which all target-specific adjacencies are accepted and the “conservative” run in which all target-specific adjacencies are broken. (*Left*) Misassembly rate between the ordered contigs, computed relative to the complete simulated sequence. (*Right*) The number of unplaced contigs (missing in the assembled chromosomes). Each data set contains approximately 5000 contigs with a mean length of 18,000 bp.

Given a resulting set of chromosomes, we call an adjacency (a pair of consecutive contigs) *correct* if these contigs have the same sign and their original positions in the target genome are adjacent (allowing jumps through unplaced fragments that were not included into the final scaffolds). Otherwise, the adjacency is called *erroneous*. For each run, we measured the error rate as the number of erroneous adjacencies divided by the total number of adjacencies. The computed error rates as well as the statistics of unplaced contigs are shown on the Figure 3. As was expected, the normal Ragout 2 strategy, which keeps a subset of target-specific adjacencies (rather than all/none of them), produces fewer errors compared with the naive strategies. RACA performance was intermediate between that of the conservative and permissive Ragout 2 strategies for the data sets with 50–250 rearrangements, while for more complex data sets, RACA produced the least accurate assemblies. The normal and conservative strategies resulted in almost the same number of unplaced contigs. However, this number was higher for the permissive strategy, which is a consequence of including chimeric adjacencies in the final scaffolds. RACA produced a higher number of unplaced contigs compared with all the Ragout 2 strategies. We additionally benchmarked Ragout 2 on the incomplete sets of references. As expected, the assemblies using all three references consistently had fewer errors compared with the assemblies using one or two closest references (for details, see Supplemental Note “Benchmarking Ragout 2 on incomplete sets of references”).

Additionally, we compared Ragout 2 and RACA using the comprehensive set of multiple human genome assemblies from Salzberg et al. (2012). On most data sets, Ragout 2 performed equally or better than RACA (for details, see Supplemental Note “Benchmarking Ragout 2 and RACA on multiple human genome assemblies”).

### Assembly of 16 laboratory mouse strains

We applied Ragout 2 to 16 mouse genomes, which included 12 laboratory strains (129S1/SvimJ, A/J, AKR/J, BALB/cJ, C3H/HeJ, C57BL/6NJ, CBA/J, DBA/2J, FVB/NJ, LP/J, NOD/ShiLtJ, NZO/H1LtJ) and four wild-derived strains (WSB/EiJ, PWK/PhJ, CAST/EiJ, SPRET/EiJ) (Lilue et al. 2018). The initial Illumina sequencing libraries (40×–70×) were assembled using SGA (Simpson and Durbin 2012). The contigs N50 ranged from 5 kb (PWK/PhJ) to 26 kb (AKR/J). Scaffolding was performed with SOAPdenovo2 (Luo et al. 2012) using multiple paired-read and mate-pair libraries with insert sizes 3, 6, and 10 kb; 40-kb fosmid ends for eight strains- and BAC ends for NOD/ShiLtJ. The improved scaffold N50 ranged from 231 kb (FVB/NJ) to 1575 kb (LP/J). Finally, the three most divergent genomes (PWK/PhJ, SPRET/EiJ, and CAST/EiJ) were additionally scaffolded using the Dovetail Genomics Chicago libraries (Putnam et al. 2016), which gave a significant contiguity improvement. The final scaffold N50 ranged from 20 Mb for SPRET/EiJ to 25 Mb for PWK/PhJ. Detailed information about the initial assembly is given in the Supplemental Note “Additional information about 16 laboratory mouse strain assembly statistics” and by Lilue et al. (2018).

For the Ragout 2 assembly, we chose the C57BL/6J *Mus musculus* strain as a single reference, as all target genomes have the same karyotype and show a good structural similarity with the C57BL/6J reference: On average, 254 adjacent synteny block pairs >10 kb from the target NGS assemblies were not adjacent in the C57BL/6J reference (which correspond to large putative rearrangements). Ragout 2 was run in three iterations with the default synteny block scale for mammalian assemblies (10,000, 500, 100). Also, see the Methods section “Synteny Block Size Selection” for an extended discussion. For each target strain, Ragout 2 produced a complete set of full-length chromosomes with the expected large-scale structure. Some assemblies also included short unlocalized fragments (homologous to the corresponding sequences in C57BL/6J) or mitochondrial (Mt) chromosomes. The statistics of assembly results are given in Table 1. The unplaced sequence for each assembly comprised <5% of the total length. We also estimated the number of missing exons as <2% for each assembly (see below). On average, 49 adjacencies between synteny blocks >10 kb from the assembled chromosomes were not present in the C57BL/6J reference (excluding the three most divergent genomes).

**Table 1.**
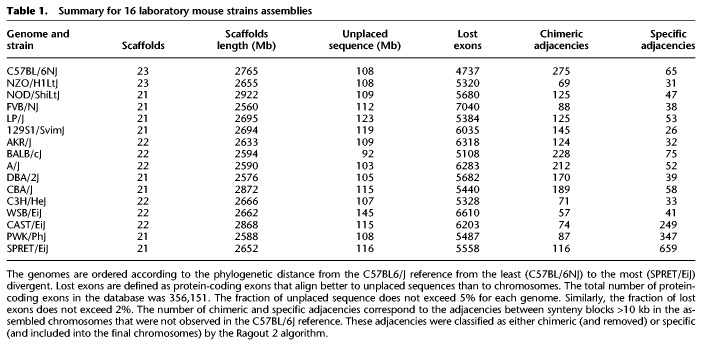
Summary for 16 laboratory mouse strains assemblies

**Figure 4.**
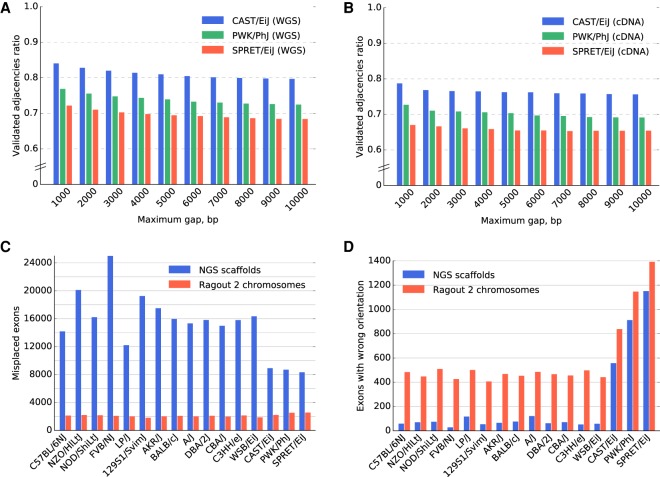
Sixteen mouse laboratory strain assemblies validation. (*A*,*B*) The validated adjacency ratio using PacBio reads depending on the maximum gap size of an adjacency for the three most divergent strains. (*A*) Whole-genome sequence data with approximately 0.5×–1× genome coverage. (*B*) Whole-exome sequencing with approximately 0.3× genome coverage. The probability of a correct and covered adjacency without a gap not being validated by the reads of length 3000 bp at 1× coverage could be estimated as 15%. (*C*,*D*) Ensembl/GENCODE transcript consistency analysis. The genomes are ordered according to the phylogenetic distance from the C57BL6/J reference from the least (C57BL/6NJ) to the most (SPRET/EiJ) divergent. (*C*) Number of exons found on nonprimary scaffolds/chromosomes. (*D*) The number of exons on the primary chromosome in a wrong orientation. The total number of transcripts in the database was 78,653. The control analysis of C57BL/6J reference genome yielded 1638 misplaced exons and 517 exons in the wrong orientation (due to ambiguous alignments of short exons). The three most divergent genomes were scaffolded using the Dovetail technology, which explains the increased number of exons with wrong orientation in the NGS scaffolds. Interestingly, the difference between the number of exons with wrong orientation between Ragout 2 chromosomes and NGS scaffolds was lower for the Dovetail-scaffolded genomes.

To estimate the structural accuracy of the assembled chromosomes, we used multiple sets of PacBio reads that were available for the three most divergent genomes (PWK/PhJ, SPRET/EiJ, and CAST/EiJ). The samples included whole-genome sequence data at approximately 0.5–1× coverage and mean read length of ∼3000 bp, as well as cDNA sequencing data from liver and spleen for each of the three strains. We classified each adjacency (a pair of consecutive NGS fragments on a Ragout 2 chromosome) as *covered* if both fragments have read alignments of at least 500 bp or as *uncovered* otherwise (47%, 43%, and 55% adjacencies were covered for the PWK/PhJ, SPRET/EiJ, and CAST/EiJ genomes, respectively). We call an adjacency *validated* if there is at least one read that has alignments >500 bp on both parts of the adjacency with a correct orientation. We then calculated the validated adjacency ratio as the number of validated adjacencies divided by the number of covered adjacencies (Fig. 4A,B show the validated adjacency ratio as a function of the maximum gap size of an adjacency). As expected, longer gaps were harder to validate as the chance of being covered with a single read decreases. Genomes that were closer to the C57BL/6J reference had more validated adjacencies, which is explained by the increasing structural divergence between the reference and the target assembly. Additionally, since the PacBio cDNA data had lower coverage, fewer adjacencies could have been validated using that data set. The probability of a correct and covered adjacency without a gap not being validated by the reads of length 3000 kb at 1× coverage could be estimated as 15%, which was in agreement with the experimental data. Additional details about this benchmark are given in Supplemental Note “Additional information about adjacency validation using PacBio reads.”

To further investigate the accuracy of coding sequence, we performed a transcript consistency analysis using the Ensembl/GENCODE comprehensive transcript set (GENCODE version M4/Ensembl 78) (Flicek et al. 2011). We aligned 356,151 protein-coding exons from the database on the combination of Ragout 2 chromosomes and corresponding unplaced sequences using BWA (leaving only the best hit for each exon) (Li and Durbin 2009). We defined *lost* exons as exons that align better to the unplaced sequence than to chromosomes (see Table 1). Then, for each of 78,378 transcripts, we determined its *primary chromosome* and *primary orientation* with respect to the alignments of the majority of its exons. Exons that align to a nonprimary chromosome of the corresponding transcript are called *misplaced*. Similarly, we calculated exons with *incorrect orientation* among the exons that align to the primary chromosome. Exons that belong to multiple transcripts were counted once. The results are shown in Figure 4, C and D. As expected, NGS assemblies had significantly higher numbers of misplaced exons than Ragout 2 chromosomes (15,519 and 2116 on average, respectively). The average number of exons with wrong orientation over all genomes was 69 for NGS assemblies and 469 for Ragout 2 (excluding PWK/PhJ, SPRET/EiJ, and CAST/EiJ). To estimate the specificity of the described benchmark, we also ran it on the C57BL/6J reference (false-positive calls may happen because of ambiguous alignments or annotation errors); the number of misplaced exons was 1638, while the number of exons with wrong orientation was 517. Thus, both statistics for Ragout 2 chromosomes are only slightly higher or equal to ones calculated for the reference, which confirms the high accuracy of the reconstruction of coding sequence.

Finally, we benchmarked the ability of Ragout 2 to preserve target-specific rearrangements that are not observed in reference genomes. We used 688 PCR primer pairs available from previous studies (Yalcin et al. 2012) that surround structural variations in different mouse genomes (Keane et al. 2014). For each genome, we extracted primer pairs that align on a single NGS scaffold with a variation in distance with respect to the C57BL/6J reference (on average, 496 primer pairs were chosen). For each such pair, we compared the alignment distances between the NGS assembly and the Ragout 2 chromosomes. While Ragout 2 corrected many structural misassemblies (see Table 1), all these target-specific structural variations were preserved.

We also compared Ragout 2 performance against RACA using three genomes for which long PacBio reads were available *(*PWK/PhJ, SPRET/EiJ, and CAST/EiJ*).* Similarly to Ragout 2 runs, RACA runs were performed using one reference (C57BL/6J), as well as all available paired-end and mate-pair libraries. Ragout 2 chromosomes consistently showed fewer split transcripts and transcripts with wrong orientation, while the number PacBio reads with correct alignment orientations was higher (for the detailed comparison, see Supplemental Note “Comparing Ragout 2 RACA using laboratory mouse genomes”).

### *Mus caroli* and *Mus pahari* assemblies

In order to see how our method performs in assembling more distant genomes from the references, we applied Ragout 2 to the *Mus caroli* and *Mus pahari* genomes. These genomes exhibit 4% and 8% sequence divergence from *M. musculus* (which is equivalent to the human–orangutan and human–marmoset divergence, respectively). The *M. caroli* and *M. pahari* contigs were assembled from Illumina reads at 135× (*M. caroli*) and 185× (*M. pahari*) coverage and further scaffolded using multiple mate-pair libraries with up to 3-kb insertion using ALLPATHS-LG (Butler et al. 2008). The scaffolds N50 was 195 kb for *M. caroli* and 331 kb for *M. pahari*. Additionally, both genomes were further scaffolded using OpGen optical maps, which improved contiguity to 4.3 Mb for *M. caroli* and 3.6 Mb for *M. pahari* (the details of NGS assembly are given by Thybert et al. 2018).

Since *M. caroli* and *M. pahari* were evolutionarily intermediate between *M. musculus and Rattus norvegicus*, we used both of these references for the Ragout 2 assembly. Additionally, for both the *M. pahari* and *M. caroli* assemblies, we used the fragments from the other genome as an extra third reference, as they provided extra adjacencies not observed in the *M. musculus and R. norvegicus* genomes. Similar to the laboratory mouse strains assembly, Ragout 2 was run in three iterations: (10,000, 500, 100). Ragout 2 assembled *M. caroli* into 26 scaffolds (consisting of 28,486 NGS fragments), which correspond to 19 autosomes, Chromosome X, and six small, unlocalized fragments. Detailed assembly statistics are given in Table 2. The assembled *M. caroli* chromosomes do not exhibit any large inter-chromosomal rearrangements with respect to the *M. musculus* genome, which was expected from physical maps as well as chromosome paintings (Thybert et al. 2018). However, we detected a large cluster of inversions in Chromosome 17 of ∼5 Mb. In addition to this karyotype-scale variation, we detected 12 synteny block adjacencies that do not appear in any of the three reference genomes, suggesting target-specific rearrangements >10 kb. In the assembled chromosomes, 28 connections between contigs >10 kb were supported by only the *M. pahari* and/or *R. norvegicus* genomes.

**Table 2.**
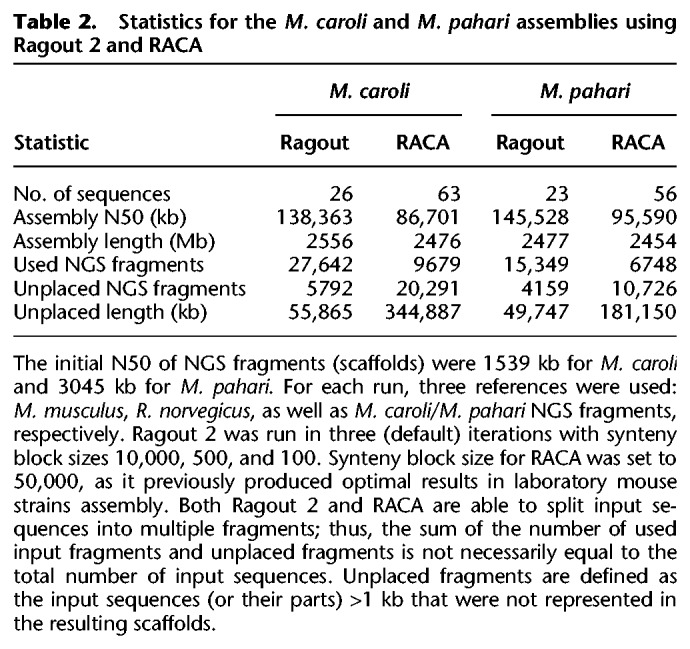
Statistics for the *M. caroli* and *M. pahari* assemblies using Ragout 2 and RACA

**Figure 5.**
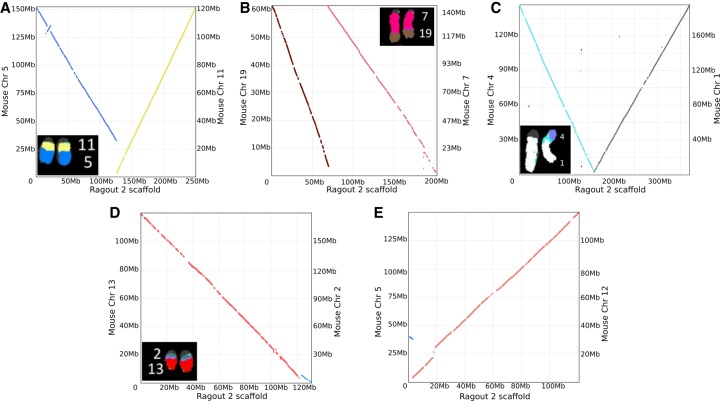
Dot-plots and corresponding chromosome paintings (*A*–*D*) showing inter-chromosomal rearrangements in *M. pahari* assembly. The rearrangement shown in *E* is supported by the *R. norvegicus* reference and might be missed from chromosome painting due to its small size (∼2 Mb). Chromosome paintings were generated by Thybert et al. (2018).

**Figure 6.**
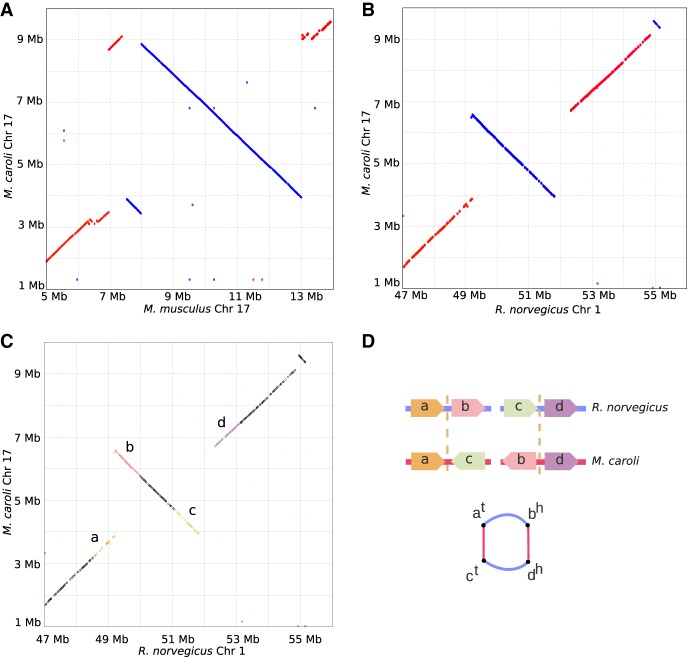
Large inversion detected in *M. caroli* Chr 17. Genome dot-plots of a Chromosome 17 region of *M. caroli* against *M. musculus* (*A*) and *R. norvegicus* (*B*) showing a cluster of structural variations of size ∼5 Mb. *M. pahari* chromosomes shows the same genomic structure. The inversion breakpoints are supported by the optical maps in both the *M. caroli* and *M. pahari* genomes. *M. musculus* and *R. norvegicus* references share one breakpoint of two different inversions. (*C*,*D*) An illustration of the chimera detection algorithm. The same inversion with highlighted synteny blocks (*C*) forms an alternating cycle of length four in the breakpoint graph (*D*). Thus, the Ragout 2 chimera detection algorithm classified these red edges as genomic (not artificial) and included the corresponding adjacencies into the assembled chromosomes.

The same protocol was used to assemble the *M. pahari* genome (see the results in Table 2). Ragout 2 reconstructed 23 scaffolds from 16,108 assembly fragments. In contrast to *M. caroli*, chromosome painting and physical mappings of *M. pahari* suggest extensive inter-chromosomal rearrangements (Thybert et al. 2018). Ragout 2 detected five chromosome fusions, four of which are consistent with the chromosome color maps and one that is supported by the *R. norvegicus* reference and might have been missed from the maps due to its relatively small size (∼2 Mb). The dot-plots of the detected large-scale rearrangements and their corresponding chromosome paintings are shown on Figure 5. Four expected chromosome fusions and 13 expected chromosome fissions were not detected by our algorithm. This was mainly caused by the missing signatures of the rearrangements in the input sequences; in particular, it is currently very hard to predict a chromosome fission if it is not supported by any of the references since target fragments cannot provide positive evidence of such an event. Ragout 2 resolves this issue by integrating physical mappings from Thybert et al. (2018). Importantly, Ragout 2 did not generate any large inter-chromosomal rearrangements that were not expected from physical mappings or references. We also detected 36 adjacencies that do not appear in any of the reference genomes, suggesting target-specific rearrangements of size >10 kb. Twenty-one connections between contigs >10 kb in the final chromosomes were supported by only the *M. caroli* and/or *R. norvegicus* genomes. We also detected a cluster of inversions with the same structure as in the *M. caroli* genome in a chromosome, homologous to a *M. musculus* Chromosome 17. The breakpoints of the detected inversions in both genomes were contained within the scaffolds generated using optical maps. *M. musculus* and *R. norvegicus* references also contain different structural variations within this region and share one inversion breakpoint (Fig. 6A,B). This might be a signature of a rearrangement hotspot (Pevzner and Tesler 2003a).

We also applied RACA to assemble *M. caroli* and *M. pahari* genomes and compared its performance against Ragout 2 (see Table 2). We used the same set of reference genomes and all available paired-end and mate-pair libraries. Synteny block size was set to 50 kb as it previously produced the optimal results on *M. musculus* genomes in terms of assembly contiguity and coverage. Some of the assembled *M. caroli* chromosomes were left fragmented (63 scaffolds, N50 = 86 Mb), and a significant portion of sequence (344 Mb) was left unplaced by RACA, which could be explained by the fact that only a single fixed synteny block size is used. In contrast, an assembly with synteny block scale of 10 kb left 63 Mb of sequence unplaced, but N50 was two times lower (40 Mb). Similarly, for the *M. pahari* assembly, RACA produced scaffolds with higher fragmentation (56 scaffolds, N50 = 96 Mb) and left more sequence unplaced (see Table 2). RACA found four of the five chromosome fusions detected by Ragout 2.

### Comparative assembly of three ape genomes

To further investigate how Ragout 2 performs in combination with multiple long-range technologies, we applied it to three recent ape genome assemblies published by Kronenberg et al. (2018). In the original study, the genomes were initially assembled using high-coverage whole-genome PacBio sequencing, which resulted in contiguous assemblies with N50 ranging from 10–12.5 Mb (detailed statistics are shown in Supplemental Note “Additional information on comparative assembly of three ape genomes”). These contigs were further ordered and oriented using various experimental data, including BioNano optical maps, Hi-C, BAC clones, and FISH. This resulted in new reference-quality assemblies of the chimpanzee, gorilla, and orangutan genomes.

Here we used Ragout 2 to assemble the initial PacBio contigs (chimpanzee: GCA_002880755.1; orangutan: GCA_002880775.1; gorilla: GCA_900006655.2) using multiple references (human, gibbon, rhesus, as well as the draft PacBio contigs). Since the PacBio assemblies were highly continuous, the synteny block size was increased to (500,000, 50,000) in comparison to the mouse assemblies. The resulting chromosome-scale assemblies were compared against the chromosomes generated by Kronenberg et al. (2018) using MashMap (Jain et al. 2017). For all three assemblies, Ragout 2 produced sets of complete chromosomes that were largely collinear with the directly assembled references (whole-genome dot-plots and additional statistics are shown in Supplemental Note “Additional information on comparative assembly of three ape genomes”). We identified nine large karyotype-scale rearrangements in gorilla, six in chimpanzee, and seven in orangutan Ragout 2 assemblies that are likely to represent misjoins. Likewise, the investigators of the original study report that they corrected three and five large-scale misassemblies in BioNano scaffolds in the orangutan and chimpanzee assemblies, respectively. The Ragout 2 misjoins were mainly caused by the unique rearrangements in the assembled genomes. The breakpoints of these rearrangements were not captured in PacBio contigs since they were flanked with long segmental duplications, unresolved by the PacBio assembler. We additionally found two smaller (<5 Mb) inversions in the gorilla and orangutan genomes. Thirteen small inversions were detected in the chimpanzee genome. However, only one of these inversions was confirmed against the RefSeq version of the chimpanzee genome (panTro4). We thus think that these small inversions do not necessarily represent Ragout 2 artifacts and require further investigation. Overall, Ragout 2 produced chromosome-scale assemblies with a number of errors comparable to the direct sequencing approaches.

## Discussion

Despite recent advances in sequencing technologies and bioinformatics algorithms, de novo assembly of a mammalian scale genome into a complete set of chromosomes remains a challenge. Many genome sequencing projects (Wang et al. 2014; Dobrynin et al. 2015; Vij et al. 2016) have used reference-guided assembly as a step in genome finishing, often followed by manual sequence curation. While multiple tools for reference-assisted assembly exist, their performance has proved limited when the reference genomes exhibit a significant number of structural variations relative to the target genome being assembled. In this paper, we have presented Ragout 2, an algorithm for chromosome assembly of large and complex genomes using multiple references. Ragout 2 joins input contigs or scaffolds into larger sequences by analyzing genome rearrangements between multiple references and the target genome. In contrast to previous approaches, Ragout 2 utilizes hierarchical synteny information, which helps to reduce gaps in the resulting chromosomes. We used simulations to show that Ragout 2 makes few errors, even in the presence of complicated rearrangements, and outperforms previous approaches in both accuracy and assembly completeness.

By using the existing *M. musculus* reference, we applied Ragout 2 to assemble 16 diverse laboratory mouse strains. Through the benchmarks, which included validations with long PacBio reads, transcriptome analysis, and PCR testing, we show that Ragout 2 produced highly accurate chromosome assemblies with <5% of sequence unplaced (2% of coding sequence). An analysis of transcript data showed a substantial improvement of the resulting assemblies from a gene structure perspective. Importantly, our algorithm is capable of preserving target-specific rearrangements, which are observed in the NGS assembly but not in the reference genomes, thus having less reference bias than simpler reference-guided approaches. We also used Ragout 2 to assemble two more challenging genomes: *M. pahari* and *M. caroli*, which exhibit major karyotype-scale differences compared with these references. *M. caroli* was assembled into a complete set of chromosomes with the expected karyotype structure. *M. pahari* chromosomes assembled by Ragout 2 contained five large intrachromosomal rearrangements, four of which were confirmed by chromosome painting techniques. The fifth rearrangement was supported by the *R. norvegicus* reference and might have been missed from chromosome maps due to its relatively small size. While Ragout 2 did not generate any unexpected rearrangements (not supported by chromosome maps or references), some expected rearrangements remained undiscovered. Most of these rearrangements were chromosome fissions, which are difficult to predict using only NGS sequencing data. Ragout 2 has a module that uses chromosome maps to guide the final assembly and successfully incorporated all expected rearrangements into the final chromosomes. We have thus shown that Ragout 2 produces accurate and complete chromosome assemblies of mammalian-scale genomes even in the presence of extensive structural rearrangement. The assembled chromosomes were used as starting points for manual sequence curation in the mouse strains assembly project (Lilue et al. 2018; Thybert et al. 2018).

The genomes of 16 laboratory mouse strains, which have been assembled using Ragout 2 with additional curations, are available from the NCBI BioProject database (https://www.ncbi.nlm.nih.gov/bioproject/) under the PRJNA310854 bio project ID. The genome assemblies of *M. caroli* and *M. pahari* were submitted to the European Nucleotide Archive (www.ebi.ac.uk/ena) and are available with accession numbers GCA_900094665.2 (*M. caroli*) and GCA_900095145.2 (*M. pahari*).

One limitation of Ragout 2 is that the algorithm currently does not support diploid genomes, thus only a single copy of each chromosome is reconstructed (possibly, as a mixture of different alleles). Complete de novo diploid genome assembly remains a challenging task, even with recent improvements in sequencing technologies, which include long Hi-C interactions or 10× read clouds that can link heterozygous variations. Being mostly orthogonal problems, the information from reference genomes could be further coupled with the direct sequencing approaches to improve de novo diploid genome assembly and phasing. While Ragout 2 uses a two-break rearrangement model to distinguish chimeric adjacencies from real rearrangements, the RACA algorithm implements a different approach in which the information from the paired-end sequencing libraries is used detect unreliable scaffold connections. The two approaches are orthogonal and ideally should be combined together in the future to get the optimal assembly results.

Many new technologies that capture long-range adjacency information are now becoming popular for de novo genome assembly (Chaisson et al. 2015; Jain et al. 2018; Kronenberg et al. 2018). The recent developments include long reads produced by PacBio and Oxford Nanopore sequencers, linked reads produced by 10x Genomics, Hi-C (Dovetail) libraries for chromosome-scale interaction maps, as well as long-range optical maps (BioNano). Currently, none of these technologies alone can provide a complete assembly of a large genome; thus, the assembly projects often employ a combination of multiple approaches followed by manual merging and curation. By analyzing three recently published ape genome assemblies, we illustrate that our comparative approach can provide a cost-efficient alternative to direct sequencing experiments and serve as an orthogonal source of adjacencies, which could be useful in misassembly detection. Importantly, the Ragout 2 algorithm does not depend on a particular sequencing or scaffolding method and thus could be easily applied to improve assemblies generated by various long-range technologies in the future.

## Methods

### Construction of synteny blocks

Ragout 2 constructs synteny blocks at multiple scales and provides a hierarchical relationship among these different scales of synteny blocks (for a complete discussion of multiscale synteny blocks, see Minkin et al. 2013; Ghiurcuta and Moret 2014). At the highest level of resolution, the alignment of multiple genomes is represented as a set of alignment blocks. Each alignment block is a set of oriented, nonoverlapping, homologous subintervals of the input genomes. To derive a set of alignment blocks, we use Cactus. Each genomic sequence *s*_*t*_ can be represented in the alphabet of alignment blocks *s*_*t*_ = *b*_*1*_, … , *b*_*n*_, where *b*_*i*_ is an alignment block of length |*b*_*i*_|. Given a set of *k* sequences *S* = {*s*_*1*_, … , *s*_*k*_ } and a parameter *minBlock*, we construct an A-Bruijn graph *G(S, minBlock)* as follows: For each alignment block *b*_*i*_ such that |*b*_*i*_| ≥ *minBlock*, we create two nodes *b*^*h*^_*i*_ and *b*^*t*^_*i*_ (representing the head and tail of each block) and connect them by a black edge. We connect heads and tails of adjacent alignment blocks in each genome s_t_ with an adjacency edge with color *C*_*t*_. The length of an adjacency edge is defined as the distance between the two alignment blocks in the corresponding genome. We also include a special infinity node (Alekseyev and Pevzner 2009) representing the ends of sequences (telomeres in complete genomes or fragment ends in draft genomes). A node is called a bifurcation if it is connected with more than one node (by colored edges) or is connected with an infinity node. A path without any bifurcation node except for its start and end is called nonbranching. Figure 1B shows an example of an A-Bruijn graph constructed from alignment blocks present in Figure 1A. Intuitively, this construction is equivalent to (1) representing each sequence in the alphabet of alignment blocks and (2) gluing together two alignment blocks if they are homologous and have size larger than *minBlock* (for a formal definition of gluing, see Medvedev et al. 2011). Constructing this A-Bruijn graph is also equivalent to constructing the breakpoint graph from multiple genomes (for a proof of equivalence, see Lin et al. 2014).

Small polymorphisms and micro-rearrangements correspond to certain cycle types or local structures in the A-Bruijn graph and disrupt large homologous regions into many shorter ones (for a thorough cycle type classification of an A-Bruijn graph, see Pham and Pevzner 2010). We use a simplification algorithm to simplify bulges and parallel paths in the A-Bruijn graph. The algorithm consists of two subprocedures—*CompressPaths* and *CollapseBulges*—and is parameterized with a value *maxGap*, which is the maximum length of the cycle/path to be simplified. *CompressPaths* is used to merge collinear alignment blocks into a larger synteny block. It starts from a random bifurcation node and traverses the graph in an arbitrary direction until it reaches another bifurcation node or an adjacency edge that is longer than *maxGap*. Then, it exchanges the traversed path with two nodes connected by a black edge (which corresponds to a new synteny block) (e.g., see Fig. 1E). A complementary procedure—*CollapseBulges*—finds all simple bulges having branch length shorter than *maxGap* in a similar manner and exchanges the bulge with a single synteny block as described previously (see Fig. 1B). These two procedures are applied one after another multiple times until no more simplification can be done. It is easily verified that the result is invariant to the order of such operations. After the simplification step, the sequences of synteny blocks are recovered by “threading” the genomes through the graph. If the blocks require additional simplification, a larger value of *minBlock* is used to construct the new A-Bruijn graph (see Fig. 1D).

The choices of *minBlock* and *maxGap* are not independent; their scales should be in agreement with each other. Using a small value of *minBlock* with a big value of *maxGap* will lead to longer blocks with low similarity (false blocks). On the other hand, if *maxGap* is much smaller than *minBlock*, the effect of simplification will be minor. Moreover, we cannot start from big *minBlock* as the initial alignment might be highly fragmented into many small alignment blocks. As a solution for these issues, we run the simplification algorithm iteratively. Starting from a small *minBlock* and *maxGap*, we gradually increase them until we reach the target synteny block scale. The values of *minBlock* and *maxGap* were chosen empirically by comparing the final results with 2D synteny dot-plot pictures.

### Repeat resolution algorithm

Repetitive synteny blocks are defined as blocks with at least two copies in a genome. We denote a set of all instances of a single repetitive synteny block inside a genome as a repeat family. Given a repeat family *RF*, for each instance in *RF* we define context as an ordered set of at most *2b* closest synteny blocks (*b* from the left and *b* from the right, any or all of which may be repetitive as well). We call a repetitive block resolved, if there is at least one unique synteny block among these *2b* blocks. If all blocks in the context are repetitive or the context is empty, the repetitive block is called unresolved. It is expected that orthologous copies of a repeat share a similar context during genome evolution. Given two contexts *c*_*1*_ and *c*_*2*_, we define *score(c_1_, c_2_)* as the alignment score between the sequences of blocks from *c*_*1*_ and *c*_*2*_ (a match between unique/repetitive blocks adds +2/+1 to the score, respectively; mismatches and gaps are penalized by −1). Let *G*_*r*_ and *G*_*t*_ be the reference and the target genomes, respectively. First, we find a mapping between *R*_*r*_ and *R*_*t*_, which are resolved blocks from *G*_*r*_ and *G*_*t*_, respectively. We construct a full bipartite graph, where nodes from each set correspond to repetitive blocks from *R*_*r*_ and *R*_*t*_, respectively. Then for each pair of nodes *r*_*i*_, *r*_*j*_ from two sets, we put an edge with weight equal to *score(c_i_, c*_j_), where *c*_*i*_ and *c*_*j*_ are contexts of *r*_*i*_, *r*_*j*_. We then find a maximum weight matching, which corresponds to an optimal mapping between *R*_*r*_ and *R*_*t*_.

After finding the matching for resolved blocks, we apply a similar approach for unresolved ones, with an exception that one repeat block from the target assembly may match against multiple repeats from the reference. The above algorithm is extended in the case of multiple references by using a strategy similar to the progressive method for multiple sequence alignment.

### Detection of chimeric adjacencies

We denote a red edge in the breakpoint graph as *supported* if there is at least one parallel reference edge in the graph; otherwise, the red edge is denoted *unsupported*. We call an adjacency *genomic* if it is a true adjacency present in the target genome and *artificial* if it is a result of a chimeric contig. Supported red edges are unlikely to be artificial; however, an unsupported red edge could be either genomic (coming from a rearrangement specific to the target genome) or artificial (in case of misassemblies). To classify each red edge as either genomic or artificial, Ragout 2 analyzes the rearrangement structure between the target genome and references. Under the *k*-break rearrangement model (Alekseyev and Pevzner 2009), any genome rearrangement (such as inversion, translocation, or chromosome fusion or fission) can be modeled as a *k*-break operation on the breakpoint graph. These operations generate cycles with multiedges of alternating multicolors (Fig. 6C,D). Thus, we expect each genomic edge to belong to an alternating cycle (including trivial cycles of length one). For each unsupported edge, if it does not belong to an alternating cycle, Ragout 2 classifies it as artificial and removes from the breakpoint graph.

### Phylogenetic tree reconstruction

When the phylogenetic tree of the input sequence is not available, it can be inferred from the adjacency information (Lin et al. 2011). Given a set of genomes represented as permutations of synteny blocks, for each pair of genomes we first build a breakpoint graph. Let *b*_*1*_ be a set of all breakpoints (graph edges) from the first genome and *b*_*2*_ from the second genome. We denote *b_1_ & b*_2_ as a set of breakpoints that are shared in both genomes. The distance between two genomes is defined as *min{size(b_1_)*, *size*(*b*_*2*_)*}*–size(*b_1_ & b_2_)*. Ragout 2 then builds a distance matrix and runs the standard neighbor-joining algorithm (Saitou and Nei 1987), which provides a good approximation of a real phylogenetic tree (Moret et al. 2001). A phylogenetic tree of human, rat, *M. pahari*, *M. caroli*, and 16 laboratory mouse genomes inferred by Ragout 2 and visualized using iTOL (Letunic and Bork 2016) is shown in Supplemental Note “Phylogenetic tree inference using Ragout 2.”

### Synteny block size selection

Synteny block scale is an important parameter for the Ragout 2 pipeline. As synteny blocks breakpoints are defined by the structural rearrangements between genomes, longer blocks represent more conserved and reliable markers. Additionally, increasing block size helps to filter out common repetitive sequence (such as SINE/LINE repeats). On the other hand, synteny blocks computed for draft assemblies might be artificially shortened because of contig fragmentation.

Through our experiments we found that blocks of size 10 kb represent a good trade-off for improving an NGS draft using reference genomes from the same species or genus. Intuitively, it is larger than typical LINE repeat size (7 kb) and covers most of the NGS contig sequence but is yet reliable enough for homologous markers comparison. The default settings for mammalian genomes additionally include two iterations with synteny block sizes 500 bp (longer than most of the of SINE repeats) and 100 bp. These additional iterations are aimed to fill assembly gaps and do not change the structure of the final chromosomes. The default synteny block size could be changed by the user. For example, one might increase it for analysis of the highly contiguous assemblies using relatively distant reference genomes.

### Iterative assembly

We first perform multiple rounds of scaffold assembly with different synteny block resolution. The scaffolds from the largest scale represent a “skeleton” for the final assembly. We then iteratively merge the existing skeleton with the set of scaffolds constructed from finer-scale synteny blocks, so as the new assembly is consistent with the skeleton structure (for details, see Kolmogorov et al. 2014). As discussed above, by default Ragout 2 is run in three iterations with synteny blocks sizes equal to 10,000, 500, and 100.

Let *S*_*skeleton*_ be a current skeleton of scaffolds and *S*_*new*_ the scaffold set that is being merged. The merging algorithm consists of two parts: rearrangement projection and gap filling. During the rearrangement projection, we first detect rearrangements between *S*_*new*_ and *S*_*skeleton*_ by constructing a two-color breakpoint graph. Nodes in this graph correspond to the contigs in *S*_*skeleton*_ and *S*_*new*_. As some target-specific rearrangements were not detected in *S*_*skeleton*_ but appear in *S*_*new*_, they will form alternating cycles on the breakpoint graph. We then apply (project) the newly detected rearrangements to the *S*_*skeleton*_. Not all rearrangements can be safely projected, because some of them might be erroneous (since smaller synteny blocks are less reliable). We call a rearrangement safe, if it (1) involves fewer than *k* breaks and (2) the chromosomal similarity before and after applying this rearrangement to *S*_*skeleton*_ is more than *c*. Chromosomal similarity is defined as the percentage of synteny blocks that stay in the same scaffold after applying the rearrangement. This prevents large chromosomal translocations, fusions, and fissions from being projected. We found that *k* = *4* and *c* = 0.9 work well in most cases. This setting also allows all common rearrangement types: inversion, transposition, small chromosomal translocation, and gene conversion. After projecting rearrangements, we insert small contigs from *S*_*new*_ to *S*_*skeleton*_, such as the resulting contig order is consistent with the order in *S*_*skeleton*_ as described by Kolmogorov et al. (2014).

### Comparing Ragout 2 and RACA using simulated human Chromosome 14 assemblies

We simulated multiple data sets with extensive structural rearrangements to benchmark the Ragout 2 algorithm and compared its performance against RACA. We took human Chromosome 14 (GRCh37/hg19 version) as an ancestral genome and chose a set of breakpoints such that they divide the chromosome into intervals of exponentially distributed length (Pevzner and Tesler 2003a), in approximate concordance with empirical data. Then, we modeled structural rearrangements (inversion, translocation, and gene conversion) with breakpoints randomly drawn from the defined breakpoint set. These rearrangements were uniformly distributed on the branches of the phylogenetic tree. Next, we modeled the NGS assembly process by fragmenting the target genome. Since each breakpoint in mammalian genomes is associated with repetitive elements (Pevzner and Tesler 2003a; Brueckner et al. 2012), we marked half of the repeats that are located near the breakpoints as “unresolved” by the assembler and fragment the genome in the corresponding positions. We further applied additional fragmentation at random positions to model the other sources of contig breaks. Finally, to mimic chimeric scaffolds generated in typical NGS studies (Kim et al. 2013), we randomly joined together 5% of the target fragments. By using this setting, we simulated three reference genomes and a target genome consisting of approximately 5000 fragments with a mean length of 18,000 bp (for the corresponding phylogenetic tree, see Supplemental Note “Benchmarking Ragout 2 on incomplete sets of references”). We then benchmarked Ragout 2 and RACA on data sets simulated as described above with different number of rearrangements (ranging from 50 to 500, which also corresponded to the breakpoint reuse rates of 5% and 50%, respectively). We note that while the newer version of human reference genome (GRCh38) is now available, our benchmarks do not depend on a particular reference build, as the synteny block reconstruction algorithm is not sensitive to slight changes in nucleotide sequence.

### Running time and memory usage

Ragout 2 requires as input a whole-genome alignment generated by Cactus (Paten et al. 2011). Generating this alignment is the most computationally demanding step of the process, taking approximately 1000 CPU hours per mammalian genome, scaling linearly with genome number, with ∼120 GB of RAM used at peak. It is typically run using an HPC cluster or on a cloud platform, such as Amazon Web Services. The next steps in the Ragout 2 pipeline do not take more than an hour for all experiments performed in this paper and can easily run on a standard desktop with 32 GB of RAM.

## Data access

The Ragout 2 package is freely available in the Supplemental Material and at http://fenderglass.github.io/Ragout/. The assemblies generated by Ragout 2 and RACA as well as the Supplemental Scripts are available at https://doi.org/10.5281/zenodo.1408269.

## Competing interest statement

S.P. and B.P. own stocks in BioTuring, Incorporated.

## Supplementary Material

Supplemental Material

## References

[GR236273KOLC1] Alekseyev MA, Pevzner PA. 2009 Breakpoint graphs and ancestral genome reconstructions. Genome Res 19: 943–957. 19218533 10.1101/gr.082784.108 PMC2675983

[GR236273KOLC2] Bradnam KR, Fass JN, Alexandrov A, Baranay P, Bechner M, Birol I, Boisvert S, Chapman JA, Chapuis G, Chikhi R, 2013 Assemblathon 2: evaluating *de novo* methods of genome assembly in three vertebrate species. Gigascience 2: 10. 23870653 10.1186/2047-217X-2-10 PMC3844414

[GR236273KOLC3] Brueckner LM, Sagulenko E, Hess EM, Zheglo D, Blumrich A, Schwab M, Savelyeva L. 2012 Genomic rearrangements at the *FRA2H* common fragile site frequently involve non-homologous recombination events across LTR and L1(LINE) repeats. Hum Genet 131: 1345–1359. 22476624 10.1007/s00439-012-1165-3

[GR236273KOLC4] Butler J, MacCallum I, Kleber M, Shlyakhter IA, Belmonte MK, Lander ES, Nusbaum C, Jaffe DB. 2008 ALLPATHS: de novo assembly of whole-genome shotgun microreads. Genome Res 18: 810–820. 18340039 10.1101/gr.7337908 PMC2336810

[GR236273KOLC5] Chaisson MJ, Huddleston J, Dennis MY, Sudmant PH, Malig M, Hormozdiari F, Antonacci F, Surti U, Sandstrom R, Boitano M, 2015 Resolving the complexity of the human genome using single-molecule sequencing. Nature 517: 608–611. 25383537 10.1038/nature13907 PMC4317254

[GR236273KOLC6] Church DM, Goodstadt L, Hillier LW, Zody MC, Goldstein S, She X, Bult CJ, Agarwala R, Cherry JL, DiCuccio M, 2009 Lineage-specific biology revealed by a finished genome assembly of the mouse. PLoS Biol 7: e1000112. 19468303 10.1371/journal.pbio.1000112 PMC2680341

[GR236273KOLC7] Dobrynin P, Liu S, Tamazian G, Xiong Z, Yurchenko AA, Krasheninnikova K, Kliver S, Schmidt-Küntzel A, Koepfli KP, Johnson W, 2015 Genomic legacy of the African cheetah, *Acinonyx jubatus*. Genome Biol 16: 277. 26653294 10.1186/s13059-015-0837-4 PMC4676127

[GR236273KOLC8] Flicek P, Amode MR, Barrell D, Beal K, Brent S, Carvalho-Silva D, Clapham P, Coates G, Fairley S, Fitzgerald S, 2011 Ensembl 2012. Nucleic Acids Res 40: D84–D90. 22086963 10.1093/nar/gkr991 PMC3245178

[GR236273KOLC9] Ghiurcuta CG, Moret BM. 2014 Evaluating synteny for improved comparative studies. Bioinformatics 30: i9–i18. 24932010 10.1093/bioinformatics/btu259 PMC4058928

[GR236273KOLC10] Gnerre S, Lander ES, Lindblad-Toh K, Jaffe DB. 2009 Assisted assembly: how to improve a *de novo* genome assembly by using related species. Genome Biol 10: R88. 19712469 10.1186/gb-2009-10-8-r88 PMC2745769

[GR236273KOLC11] Gnerre S, Lander ES, Lindblad-Toh K, Jaffe DB. 2011 High-quality draft assemblies of mammalian genomes from massively parallel sequence data. Proc Natl Acad Sci 108: 1513–1518. 21187386 10.1073/pnas.1017351108 PMC3029755

[GR236273KOLC13] Hickey G, Paten B, Earl D, Zerbino D, Haussler D. 2013 HAL: a hierarchical format for storing and analyzing multiple genome alignments. Bioinformatics 29: 1341–1342. 23505295 10.1093/bioinformatics/btt128 PMC3654707

[GR236273KOLC14] Iqbal Z, Caccamo M, Turner I, Flicek P, McVean G. 2012 *De novo* assembly and genotyping of variants using colored de Bruijn graphs. Nat Genet 44: 226–232. 22231483 10.1038/ng.1028 PMC3272472

[GR236273KOLC15] Jain C, Dilthey A, Koren S, Aluru S, Phillippy AM. 2017 A fast approximate algorithm for mapping long reads to large reference databases. In International Conference on Research in Computational Molecular Biology, pp. 66–81. Springer, Cham, Switzerland. 10.1089/cmb.2018.0036 PMC6067103 29708767

[GR236273KOLC16] Jain M, Koren S, Miga KH, Quick J, Rand AC, Sasani TA, Tyson JR, Beggs AD, Dilthey AT, Fiddes IT, 2018 Nanopore sequencing and assembly of a human genome with ultra-long reads. Nat Biotechnol 36: 338–345. 29431738 10.1038/nbt.4060 PMC5889714

[GR236273KOLC17] Jarvis ED, Mirarab S, Aberer AJ, Li B, Houde P, Li C, Ho SY, Faircloth BC, Nabholz B, Howard JT, 2014 Whole-genome analyses resolve early branches in the tree of life of modern birds. Science 346: 1320–1331. 25504713 10.1126/science.1253451 PMC4405904

[GR236273KOLC18] Keane TM, Wong K, Adams DJ, Flint J, Reymond A, Yalcin B. 2014 Identification of structural variation in mouse genomes. Front Genet 5: 192. 25071822 10.3389/fgene.2014.00192 PMC4079067

[GR236273KOLC19] Kent WJ, Baertsch R, Hinrichs A, Miller W, Haussler D. 2003 Evolution's cauldron: duplication, deletion, and rearrangement in the mouse and human genomes. Proc Natl Acad Sci 100: 11484–11489. 14500911 10.1073/pnas.1932072100 PMC208784

[GR236273KOLC20] Kim J, Larkin DM, Cai Q, Asan, Zhang Y, Ge RL, Auvil L, Capitanu B, Zhang G, Lewin HA, 2013 Reference-assisted chromosome assembly. Proc Natl Acad Sci 110: 1785–1790. 23307812 10.1073/pnas.1220349110 PMC3562798

[GR236273KOLC21] Kolmogorov M, Raney B, Paten B, Pham S. 2014 Ragout: a reference-assisted assembly tool for bacterial genomes. Bioinformatics 30: i302–i309. 24931998 10.1093/bioinformatics/btu280 PMC4058940

[GR236273KOLC22] Koren S, Phillippy AM. 2015 One chromosome, one contig: complete microbial genomes from long-read sequencing and assembly. Curr Opin Microbiol 23: 110–120. 25461581 10.1016/j.mib.2014.11.014

[GR236273KOLC23] Kronenberg ZN, Fiddes IT, Gordon D, Murali S, Cantsilieris S, Meyerson OS, Underwood JG, Nelson BJ, Chaisson MJP, Dougherty ML, 2018 High-resolution comparative analysis of great ape genomes. Science 360: eaar6343. 29880660 10.1126/science.aar6343 PMC6178954

[GR236273KOLC24] Lander ES, Linton LM, Birren B, Nusbaum C, Zody MC, Baldwin J, Devon K, Dewar K, Doyle M, FitzHugh W, 2001 Initial sequencing and analysis of the human genome. Nature 409: 860–921. 11237011 10.1038/35057062

[GR236273KOLC25] Letunic I, Bork P. 2016 Interactive tree of life (iTOL) v3: an online tool for the display and annotation of phylogenetic and other trees. Nucleic Acids Res 44: W242–W245. 27095192 10.1093/nar/gkw290 PMC4987883

[GR236273KOLC26] Li H, Durbin R. 2009 Fast and accurate short read alignment with Burrows–Wheeler transform. Bioinformatics 25: 1754–1760. 19451168 10.1093/bioinformatics/btp324 PMC2705234

[GR236273KOLC27] Lilue J, Doran AG, Fiddes IT, Abrudan M, Armstrong J, Bennett R, Chow W, Collins J, Collins S, Czechanski A, 2018 Sixteen diverse laboratory mouse reference genomes define strain-specific haplotypes and novel functional loci. Nat Genet 10.1038/s41588-018-0223-8. PMC6205630 30275530

[GR236273KOLC28] Lin Y, Rajan V, Moret BM. 2011 Fast and accurate phylogenetic reconstruction from high-resolution whole-genome data and a novel robustness estimator. J Comput Biol 18: 1131–1139. 21899420 10.1089/cmb.2011.0114

[GR236273KOLC29] Lin Y, Nurk S, Pevzner PA. 2014 What is the difference between the breakpoint graph and the de Bruijn graph? BMC Genomics 15: S6. 10.1186/1471-2164-15-S6-S6 PMC4240671 25572416

[GR236273KOLC30] Luo R, Liu B, Xie Y, Li Z, Huang W, Yuan J, He G, Chen Y, Pan Q, Liu Y, 2012 SOAPdenovo2: an empirically improved memory-efficient short-read *de novo* assembler. Gigascience 1: 18. 23587118 10.1186/2047-217X-1-18 PMC3626529

[GR236273KOLC31] Medvedev P, Pham S, Chaisson M, Tesler G, Pevzner P. 2011 Paired de Bruijn graphs: a novel approach for incorporating mate pair information into genome assemblers. J Comput Biol 18: 1625–1634. 21999285 10.1089/cmb.2011.0151 PMC3216098

[GR236273KOLC32] Minkin I, Patel A, Kolmogorov M, Vyahhi N, Pham S. 2013 Sibelia: a scalable and comprehensive synteny block generation tool for closely related microbial genomes. In International Workshop on Algorithms in Bioinformatics, pp. 215–229. Springer, Berlin Heidelberg.

[GR236273KOLC33] Moret BM, Wang LS, Warnow T, Wyman SK. 2001 New approaches for reconstructing phylogenies from gene order data. Bioinformatics 17: S165–S173. 11473006 10.1093/bioinformatics/17.suppl_1.s165

[GR236273KOLC35] Paten B, Earl D, Nguyen N, Diekhans M, Zerbino D, Haussler D. 2011 Cactus: algorithms for genome multiple sequence alignment. Genome Res 21: 1512–1528. 21665927 10.1101/gr.123356.111 PMC3166836

[GR236273KOLC36] Peng Y, Leung HCM, Yiu SM, Chin FYL. 2010 IDBA–a practical iterative de Bruijn graph de novo assembler. In Annual International Conference on Research in Computational Molecular Biology, pp. 426–440. Springer, Berlin Heidelberg.

[GR236273KOLC37] Pham SK, Pevzner PA. 2010 DRIMM-Synteny: decomposing genomes into evolutionary conserved segments. Bioinformatics 26: 2509–2516. 20736338 10.1093/bioinformatics/btq465

[GR236273KOLC38] Pevzner P, Tesler G. 2003a Human and mouse genomic sequences reveal extensive breakpoint reuse in mammalian evolution. Proc Natl Acad Sci 100: 7672–7677. 12810957 10.1073/pnas.1330369100 PMC164646

[GR236273KOLC39] Pevzner P, Tesler G. 2003b Genome rearrangements in mammalian evolution: lessons from human and mouse genomes. Genome Res 13: 37–45. 12529304 10.1101/gr.757503 PMC430962

[GR236273KOLC40] Pontius JU, Mullikin JC, Smith DR; Agencourt Sequencing Team, Lindblad-Toh K, Gnerre S, Clamp M, Chang J, Stephens R, Neelam B, 2007 Initial sequence and comparative analysis of the cat genome. Genome Res 17: 1675–1689. 17975172 10.1101/gr.6380007 PMC2045150

[GR236273KOLC41] Putnam NH, O'Connell BL, Stites JC, Rice BJ, Blanchette M, Calef R, Troll CJ, Fields A, Hartley PD, Sugnet CW, 2016 Chromosome-scale shotgun assembly using an in vitro method for long-range linkage. Genome Res 26: 342–350. 26848124 10.1101/gr.193474.115 PMC4772016

[GR236273KOLC42] Richter DC, Schuster SC, Huson DH. 2007 OSLay: optimal syntenic layout of unfinished assemblies. Bioinformatics 23: 1573–1579. 17463020 10.1093/bioinformatics/btm153

[GR236273KOLC43] Rissman AI, Mau B, Biehl BS, Darling AE, Glasner JD, Perna NT. 2009 Reordering contigs of draft genomes using the Mauve Aligner. Bioinformatics 25: 2071–2073. 19515959 10.1093/bioinformatics/btp356 PMC2723005

[GR236273KOLC44] Saitou N, Nei M. 1987 The neighbor-joining method: a new method for reconstructing phylogenetic trees. Mol Biol Evol 4: 406–425. 3447015 10.1093/oxfordjournals.molbev.a040454

[GR236273KOLC45] Salzberg SL, Phillippy AM, Zimin A, Puiu D, Magoc T, Koren S, Treangen TJ, Schatz MC, Delcher AL, Roberts M, 2012 GAGE: a critical evaluation of genome assemblies and assembly algorithms. Genome Res 22: 557–567. 22147368 10.1101/gr.131383.111 PMC3290791

[GR236273KOLC46] Scally A, Dutheil JY, Hillier LW, Jordan GE, Goodhead I, Herrero J, Hobolth A, Lappalainen T, Mailund T, Marques-Bonet T, 2012 Insights into hominid evolution from the gorilla genome sequence. Nature 483: 169–175. 22398555 10.1038/nature10842 PMC3303130

[GR236273KOLC47] Simpson JT, Durbin R. 2012 Efficient de novo assembly of large genomes using compressed data structures. Genome Res 22: 549–556. 22156294 10.1101/gr.126953.111 PMC3290790

[GR236273KOLC48] Simpson JT, Wong K, Jackman SD, Schein JE, Jones SJ, Birol I. 2009 ABySS: a parallel assembler for short read sequence data. Genome Res 19: 1117–1123. 19251739 10.1101/gr.089532.108 PMC2694472

[GR236273KOLC49] Thybert D, Roller M, Navarro FCP, Fiddes I, Streeter I, Feig C, Martin-Galvez D, Kolmogorov M, Janoušek V, Akanni W, 2018 Repeat associated mechanisms of genome evolution and function revealed by the *Mus caroli* and *Mus pahari* genomes. Genome Res 28: 448–459. 29563166 10.1101/gr.234096.117 PMC5880236

[GR236273KOLC50] Venter JC, Adams MD, Myers EW, Li PW, Mural RJ, Sutton GG, Smith HO, Yandell M, Evans CA, Holt RA, 2001 The sequence of the human genome. Science 291: 1304–1351. 11181995 10.1126/science.1058040

[GR236273KOLC51] Vij S, Kuhl H, Kuznetsova IS, Komissarov A, Yurchenko AA, Van Heusden P, Singh S, Thevasagayam NM, Prakki SR, Purushothaman K, 2016 Chromosomal-level assembly of the Asian seabass genome using long sequence reads and multi-layered scaffolding. PLoS Genet 12: e1005954. 27082250 10.1371/journal.pgen.1005954 PMC4833346

[GR236273KOLC52] Wang B, Ekblom R, Bunikis I, Siitari H, Höglund J. 2014 Whole genome sequencing of the black grouse (*Tetrao tetrix*): reference guided assembly suggests faster-Z and MHC evolution. BMC Genomics 15: 180. 24602261 10.1186/1471-2164-15-180 PMC4022176

[GR236273KOLC53] Yalcin B, Wong K, Bhomra A, Goodson M, Keane TM, Adams DJ, Flint J. 2012 The fine-scale architecture of structural variants in 17 mouse genomes. Genome Biol 13: R18. 22439878 10.1186/gb-2012-13-3-r18 PMC3439969

[GR236273KOLC54] Zerbino DR, Birney E. 2008 Velvet: algorithms for de novo short read assembly using de Bruijn graphs. Genome Res 18: 821–829. 18349386 10.1101/gr.074492.107 PMC2336801

