# Supplementary material for: Chromosome assembly of large and complex genomes using multiple references
Source: Genome Res. 2018 Nov;28(11):1720–32. doi: 10.1101/gr.236273.118 (PMC6211643; doi:10.1101/gr.236273.118)
Supplement: Supplemental Material [file supp_28_11_1720__index.html]

Chromosome assembly of large and complex genomes using multiple references — Supplemental Material 

# Chromosome assembly of large and complex genomes using multiple references

## Supplemental Material

- Supplemental\_Material.pdf
- Supplemental\_Code.zip
