# Supplementary material for: Chromosome assembly of large and complex genomes using multiple references
Source: Genome Res. 2018 Nov;28(11):1720–32. doi: 10.1101/gr.236273.118 (PMC6211643; doi:10.1101/gr.236273.118)
Supplement: Supplemental Material [file supp_gr.236273.118_Supplemental_Material.pdf]

Supplemental Materials

Chromosome assembly of large and complex genomes using multiple references

Mikhail Kolmogorov[1], Joel Armstrong[2], Brian J. Raney[2], Ian Streeter[3], Matthew Dunn[4], Fengtang Yang[4], Duncan Odom[4,5], Paul Flicek[3,4], Thomas Keane[4], David Thybert*[3,6], Benedict Paten*[2] , and Son Pham*[7]

[1] Department of Computer Science and Engineering, University of California San Diego, USA
[2] Center for Biomolecular Science and Engineering, University of California Santa Cruz, USA
[3] European Molecular Biology Laboratory, European Bioinformatics Institute, Wellcome Genome Campus, Hinxton CB10 1SD, UK
[4] Wellcome Trust Sanger Institute, Wellcome Genome Campus, Hinxton CB10 1SA, UK
[5] Cancer Research UK Cambridge Institute, University of Cambridge, CB2 0RE Cambridge UK;
[6]Earlham Institute, Norwich Research Park, Norwich NR4 7UG, UK
[7]BioTuring Inc., San Diego, California, USA[1*]

Table of contents

1. Benchmarking Ragout 2 on incomplete sets of references
2. Benchmarking Ragout 2 and RACA on multiple human genome assemblies
3. Additional information about 16 laboratory mouse strain assembly statistics
4. Additional information about adjacency validation using PacBio reads
5. Comparing Ragout 2 RACA using laboratory mouse genomes
6. Additional information on comparative assembly of three ape genomes
7. Phylogenetic tree inference using Ragout 2

[1]* Corresponding authors
Address correspondence to: Dr. Son Pham, sonpham@bioturing.com

1. Benchmarking Ragout 2 on incomplete sets of references.

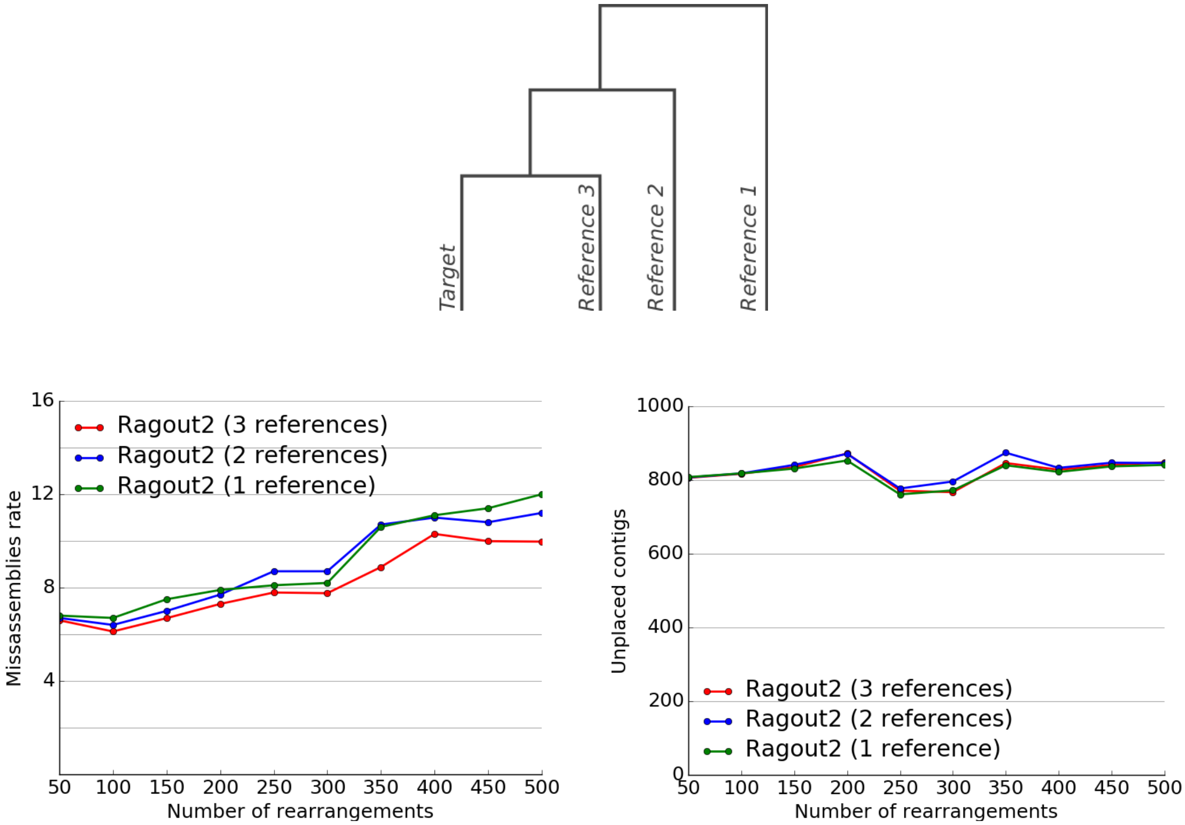

Supplementary Figure 1. Statistics of Ragout 2 assemblies using incomplete sets of references. (Top) Phylogenetic tree of the simulated reference genomes. Ragout 2 was benchmarked using one, two or three closest references. (Bottom left) Misassembly rates depending on the number of references. The rates were computed using the method described in the simulated datasets section. As it was expected, the assemblies with three references were consistently more accurate than the assemblies with two and one references, since each additional genome provides unique adjacency information. (Bottom right) The number of unplaced contigs did not show any correlation with the number of reference genomes. The total length of unplaced contigs does not exceed 4 Mb.

2. Benchmarking Ragout 2 and RACA on multiple human genome assemblies

We benchmarked the performance of the Ragout 2 and RACA algorithms using the multiple human Chromosome 14 assemblies that were previously generated by different NGS assemblers as a part of the GAGE assembly evaluation study [Salzberg et al., 2012]. The scaffolding results for RACA were taken from the original manuscript [Kim et al, 2013]. Similarly to the previous benchmarks, we selected 50 kb synteny block resolution as the most optimal for all assemblies except SGA, for which 10 kb was chosen. Similarly, we used *M. musculus* and *P. abelii* references to assemble the NGS contigs into chromosomes using Ragout 2. We evaluated accuracy of the assemblies by aligning them on the reference chromosome using LASTZ [Harris, 2007] and calculating the number of breakpoints between alignments with inconsistent order / orientation. The misassembly rates were computed on two scales: 5 kb and 50 kb. The results are given in the Supplementary Table 1. The error rates for both tools were highly correlated across the datasets, confirming that the choice of initial contigs and a reference genome is critical for the reference chromosome assembly. On most of the datasets Ragout 2 performed equally or better than RACA in terms of N50 and structural accuracy.

Dataset	NGS fragments N50 (Mb)	Ragout 2 N50 (Mb)	RACA N50 (Mb)	Ragout 2 errors	RACA errors
ALLPATHS-LG + mouse ref.	81	55	58	1 (0)	3 (2)
ALLPATHS-LG + orang. ref.		83	86	0 (0)	1 (0)
Bambus2 + mouse ref.	0.3	98	26	72 (1)	59 (2)
Bambus2 + orang. ref.		95	72	61 (3)	99 (0)
CABOG + mouse ref.	0.4	86	60	3 (5)	17 (5)
CABOG + orang. ref.		86	81	0 (0)	6 (6)
MSR-CA + mouse ref.	0.9	72	55	12 (4)	53 (4)
MSR-CA + orang. ref.		89	28	31 (0)	60 (0)
SGA + mouse ref.	0.07	73	47	12 (4)	11 (0)
SGA + orang. ref.		88	77	0 (0)	4 (0)
SOAPdenovo + mouse ref.	0.4	100	53	9 (0)	1 (0)
SOAPdenovo + orang. ref.		97	84	0 (0)	6 (0)
Velvet + mouse ref.	0.8	152	89	88 (0)	97 (0)
Velvet + orang. ref.		104	123	145 (0)	166 (0)

Supplementary Table 1. **Comparison of Ragout 2 and RACA using the multiple human Chromosome 14 assemblies from the GAGE dataset.** The misassembly errors were computed for alignments of size more than 5 kb (error rates for alignments longer than 50 kb are shown in brackets).

3. Additional information about 16 laboratory mouse strain assembly statistics.

Genome and Strain	Total length (Mb)	Contigs N50 (kb)	Scaffolds N50 (kb)	Ragout 2 chr. N50 (kb)	Unplaced length (kb)
C57BL/6NJ	2,781	13	1,402	138,565	37,099
NZO/H1LtJ	2,704	13	833	149,192	34,871
NOD/ShiLtJ	2,943	10	833	161,192	38,312
FVB/NJ	2,557	18	231	144,449	31,154
LP/J	2,704	14	1,575	151,069	26,422
129S1/SvimJ	2,695	16	499	134,859	37,099
AKR/J	2,699	26	663	150,078	42,75
BALB/cJ	2,597	18	729	145,931	29,869
A/J	2,593	21	803	146,074	36,3
DBA/2J	2,578	15	715	144,105	27,3
CBA/J	2,884	14	1,024	144,460	36,929
C3H/HeJ	2,672	16	780	149,720	2,047
WSB/EiJ	2,671	13	783	132,947	17,809
CAST/EiJ	2,635	13	24,445	133,863	18,916
PWK/PhJ	2,533	5	24,747	129,011	26,179
SPRET/EiJ	2,593	19	19,680	132,838	32,561

Supplementary Table 2. Assembly statistics for 16 laboratory mouse strains. The genomes are ordered according to the phylogenetic distance from the C57BL6/J reference from the least (C57BL/6NJ) to the most (SPRET/EiJ) divergent. The initial NGS assembly was performed using SGA. Scaffolding was performed with SOAPdenovo2 using multiple paired-read and mate-pair libraries with insert sizes 3, 6 and 10 kb, 40 kb fosmid ends for eight strains, and BAC ends for NOD/ShiLtJ. Additionally, for the three most divergent genomes (PWK/PhJ, SPRET/EiJ and CAST/EiJ) scaffolding based on a Dovetail protocol was applied. The library accession number are reported in [Lilue et al., 2018].

4. Additional information about adjacency validation using PacBio reads

PacBio alignments were performed using BLASR with the default parameters. We selected 500bp as a minimum alignment size because is long enough to exclude most of the SINE matches (typically 80-500bp), but also short enough to provide better adjacency coverage.

Given the genome of size 3 GB and the reads of length 3,000 bp at 1× coverage, we estimated the probability of a region of length 500 bp (1,000 bp) is covered in full by at least one read as 56% (51%) through the direct modeling of read sampling. Thus, the probability of a correct and covered adjacency without a gap not validated by any reads could be computed as :

Prob(left flanking region of size 500 bp is covered) x
Prob(right flanking region of size 500 bp is covered) x
Prob(adjacency region of 1000bp is not covered) ≈15 %

5. Comparing Ragout 2 RACA using laboratory mouse genomes

We compared Ragout 2 performance against RACA using three mouse genomes for which long PacBio reads were available (*PWK/PhJ, SPRET/EiJ and CAST/EiJ).* Similarly to Ragout 2, RACA runs were performed using one reference (*C57BL/6J*) as well as all available paired-end and mate-pair libraries. To select an optimal synteny block resolution, each genome was assembled multiple times with different minimum block size threshold: 10 kb, 50 kb and 150 kb (as in the original manuscript [Kim et al, 2013]). The detailed statistics are given in Supplementary Table 3. The assemblies produced by RACA had the expected chromosome structure for the larger block sizes (50 kb and 150 kb), but the assemblies with the block size of 10 kb were more fragmented. As expected, the total length of unplaced sequence was also positively correlated with increase of the synteny blocks scale. All RACA assemblies had more unplaced sequence and lost exons than the corresponding Ragout assemblies, which highlights the benefits of iterative assembly with multiple synteny block scales. We then used the GENCODE gencode transcript set to compare the structural accuracy of Ragout 2 and RACA chromosomes (see Supplementary Table 2). Ragout chromosomes consistently showed less split transcripts and transcripts with wrong orientation, while the number PacBio reads with correct alignment orientations was higher.

Dataset	Scaffolds	Scaffolds length (Mb)	N50 (Mb)	Unplaced (Mb)	Lost exons
CAST/EiJ, 10 kb	37	2,552	115	141	8,650
CAST/EiJ, 50 kb	20	2,544	131	150	9,707
CAST/EiJ, 150 kb	21	2,536	128	158	10,383
PWK/PhJ, 10 kb	48	2,454	95	129	9,742
PWK/PhJ, 50 kb	22	2,360	124	223	21,143
PWK/PhJ, 150 kb	19	2,356	128	227	21,402
SPRET/EiJ, 10 kb	44	2,536	97	132	8,080
SPRET/EiJ, 50 kb	23	2,536	128	132	7,968
SPRET/EiJ, 150 kb	20	2,536	130	132	8,538

Supplementary Table 3. Statistics for RACA assemblies of PWK/PhJ, SPRET/EiJ and CAST/EiJ*.* Each genome was assembled using three different synteny block scales.

Dataset	Missplaced exons, #RACA - #Ragout 2	Exons with wrong orientation, #RACA - #Ragout 2	Consistent PacBio reads, #Ragout 2 - #RACA
CAST/EiJ, 10 kb	1,457	484	3,386
CAST/EiJ, 50 kb	1,211	470	4,225
CAST/EiJ, 150 kb	1,460	529	2,196
PWK/PhJ, 10 kb	1,762	494	1,494
PWK/PhJ, 50 kb	1,441	627	6,127
PWK/PhJ, 150 kb	1,359	625	6,145
SPRET/EiJ, 10 kb	1,559	423	1,809
SPRET/EiJ, 50 kb	1,553	609	1,444
SPRET/EiJ, 150 kb	1,489	568	934

Supplementary Table 4. Structural accuracy comparison of RACA and Ragout 2 chromosomes. All statistics are given as differences between the corresponding values calculated for RACA and Ragout 2 results. Consistent PacBio reads were defined as reads that have all local alignments with the same orientation.

6. Additional information on comparative assembly of three ape genomes

Statistic	Gorilla	Chimpanzee	Orangutan
PacBio contigs N50 (Mb)	10	12.4	11
Ragout chr. N50 (Mb)	151	144	133
Total chr. length (Mb)	2,894	2,764	2,839
Total unplaced length (Mb)	375	276	282
Number of chr. collinear with [Kronenberg et al., 2018]	17	16	17
Large SV against [Kronenberg et al., 2018]	3 fusions<br>6 inversions	1 fusion<br>5 inversions	1 fusion<br>6 inversions
1 to 5 Mb SV against [Kronenberg et al., 2018]	2 inversions<br>(1 against gorGor4)	13 inversions<br>(1 against panTro4)	2 inversions<br>(0 against ponAbe2)

Supplementary Table 5. Ragout 2 assemblies of three ape genomes. Ragout 2 was used to assemble PacBio contigs published by [Kronenberg et al., 2018]. The assemblies were generated using three complete references (human, gibbon and rhesus) as well as draft PacBio contigs. Resulting scaffolds were compared against the chromosomes generated by [Kronenberg et al., 2018]. The authors of the original study reported that they corrected three and five large-scale misassemblies in BioNano scaffolds in orangutan and chimpanzee assemblies, respectively. All large SVs, but not all small (1-5 Mb) SVs were also confirmed against the RefSeq genome versions.

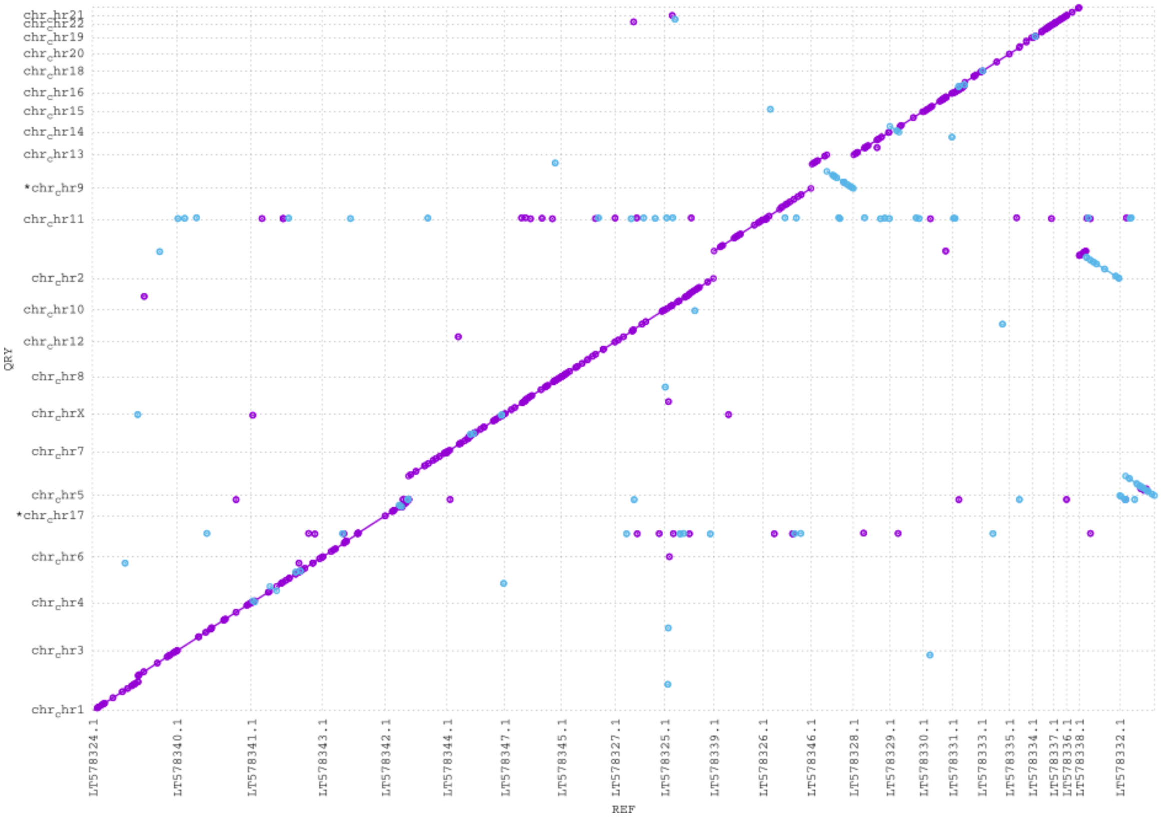

A. Gorilla chromosomes

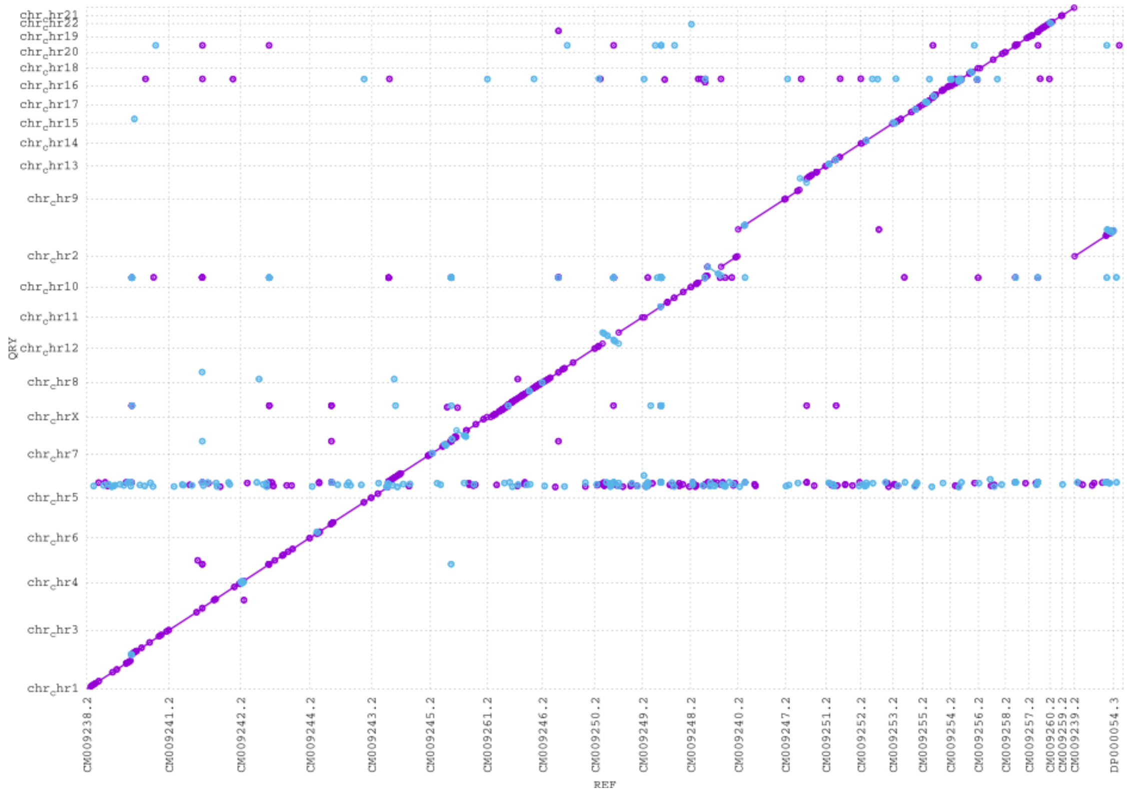

B. Chimpanzee chromosomes

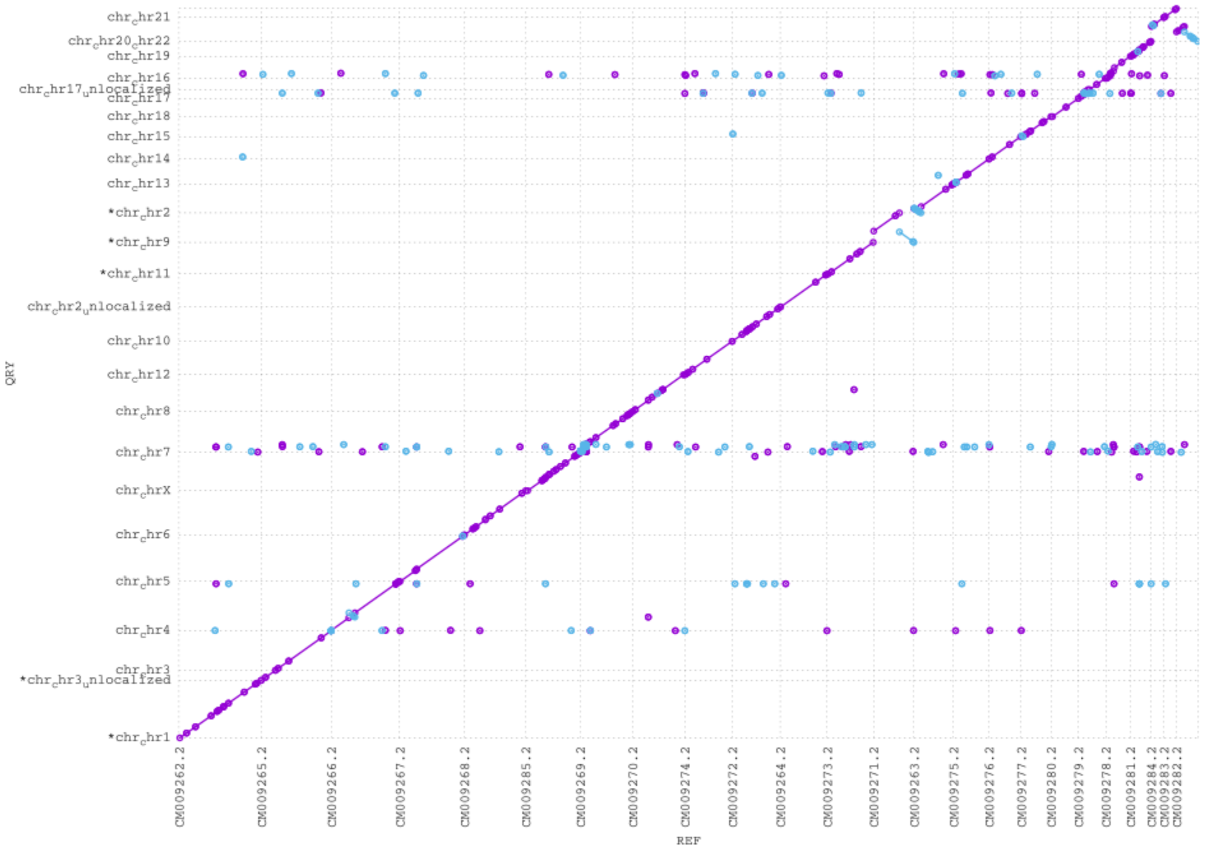

C. Orangutan chromosomes

Supplementary Figure 2. Full-genome dot-plots of Ragout 2 chromosomes against the chromosomes produced in [Kronenberg et al., 2018]. Dot-plots were computed using MashMap. Each rectangle corresponds to a pairwise chromosome mapping. Purple and blue lines represent alignments of the same or reverse complement strands, respectively. The alignment endpoints are marked with dots of the corresponding color.

7. Phylogenetic tree inference using Ragout 2

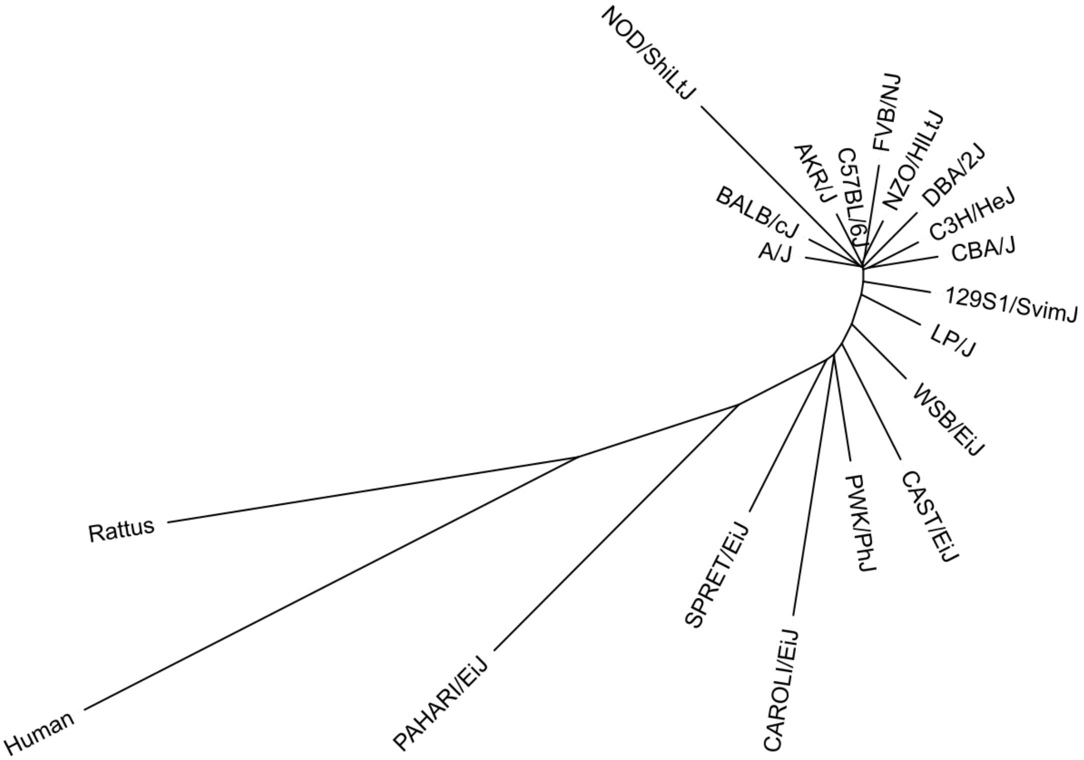

Supplementary Figure 3. Phylogenetic tree inference using Ragout 2. A phylogenetic tree of human, rat, *M. pahari, M. caroli,* and 16 laboratory mouse genomes inferred by Ragout 2 based on the breakpoint distances between the genomes. The distances were computed using the finest synteny block scale (100) to incorporate more adjacency information. The tree is largely consistent with the tree that could be inferred from mitochondrial sequence using the standard methods with an exception that *M. caroli* was slightly misplaced because of the decrease of chromosome rearrangement activity that occurred within *M. musculus* - *M. caroli* branch compared to the ancestral branch including Mus pahari [Thybert et al. 2018]. The tree was visualized using iTOL [Letunic et al., 2016].
